# Uncovering Spatiotemporal and Functional Dynamics of Long Non-coding RNAs During Alzheimer’s Progression in the Human Brain at Single-Cell Resolution

**DOI:** 10.1007/s12035-026-05859-z

**Published:** 2026-04-30

**Authors:** Allan de Carvalho, Izabela Mamede, Leonardo Sanches, Guilherme Juvenal, Fernanda Tibolla Viero, Gloria Regina Franco, Yong Tang, Eduardo Moraes Reis, Henning Ulrich

**Affiliations:** 1 Department of Biochemistry, Institute of Chemistry, University of São Paulo, São Paulo, SP Brazil; 2 Department of Biochemistry and Molecular Biology, Federal University of Viçosa, Viçosa, MG Brazil; 3 Department of Biochemistry, Institute of Biological Sciences, Federal University of Minas Gerais, Belo Horizonte, MG Brazil; 4 International Joint Research Centre On Purinergic Signalling, Chengdu University of Traditional Chinese Medicine, Chengdu, 611137 China; 5 School of Health and Rehabilitation, Chengdu University of Traditional Chinese Medicine, Chengdu, 611137 China

**Keywords:** SnRNA-seq, Long noncoding RNA, *Cis*-acting RNA, Neurodegeneration, Neural circuitry, Cerebral cortex

## Abstract

Increasing prevalence of Alzheimer’s disease, driven by population aging, highlights the need to investigate its underlying molecular mechanisms. Within this context, long non-coding RNAs (lncRNAs) have emerged as a key regulatory layer. To advance the understanding of lncRNA dysregulation and function during Alzheimer’s disease progression, we reanalyzed publicly available single-nucleus RNA sequencing (snRNA-seq) datasets. The selected transcriptomic datasets were integrated and subjected to differential expression and genomic co-localization correlation analyses to infer putative *cis*-regulatory mechanisms. Our results reveal conserved cell-type composition and a shared transcriptional trajectory across brain regions during Alzheimer’s disease progression. In contrast, lncRNAs displayed marked cell-type and context specificity and formed coordinated expression patterns with neighboring genes within defined chromatin contexts. These associations suggest potential *cis*-regulatory roles and implicate lncRNAs in processes such as synaptic plasticity and maladaptive oligodendrocyte differentiation linked to myelin dysfunction. While these findings are primarily hypothesis-generating, they provide a cross-regional framework and a prioritized set of candidate lncRNAs for future functional investigation in Alzheimer’s disease.

**Supplementary Information:**

The online version contains supplementary material available at 10.1007/s12035-026-05859-z.

## Background

Alzheimer’s Disease (AD) is a chronic neurodegenerative condition that affects more than 50 million people worldwide and stands as the leading cause of cognitive decline and dementia [1]. The molecular profile of AD is characterized by the dysregulation of hundreds of genes in several biological processes, such as neural-immune system maintenance, synaptic function, and metabolism, especially in cortical brain cells [2].

The most accepted hypothesis explaining Alzheimer’s neurodegeneration centers on the beta-amyloid (Aβ) cascade, in which the Amyloid Precursor Protein (APP) cleavage pathway leads to the abnormal accumulation of Aβ [3]. However, it is known that other processes also occur in parallel, such as neuroinflammation, blood–brain barrier breakdown, synaptic loss, and the hyperphosphorylation of microtubule-associated protein Tau (MAPT) [4]. Tau pathology has special importance since the phosphorylated Tau protein forms aggregates called neurofibrillary tangles (NFTs), which support the disease staging. That is, according to the spread profile of NFTs, we can classify AD progression into six levels, known as Braak stages [5].

Notably, AD pathology causes molecular and cellular alterations even before any clinical symptoms, which are more prevalent from Braak stage II on [6]. The noncoding transcriptome, especially long noncoding RNAs (lncRNAs), has been widely investigated as one of the most important gene expression regulators in the Central Nervous System (CNS) context [7, 8]. Acting as both *trans* and *cis-regulators* of gene expression, these molecules are essential for thousands of physiological and pathological processes [9]. *Cis*-acting lncRNAs, which compose a substantial fraction of lncRNAs in the cell nucleus, modulate gene expression dynamics by diverse mechanisms, such as recruitment of chromatin remodeling proteins and targeting DNA regulatory loci [10].

Concerning AD pathology, most studies have focused on individual lncRNA interactions with known targets involved in the disease. For example, *BACE1-AS* (*BACE1* Antisense RNA) is an intronic antisense transcript produced by the gene locus of β-secretase, the enzyme that promotes the cleavage of APP in the Aβ pathway [11]. This approach also fails in elucidating the heterogeneous expression of lncRNAs within cell subtypes, which has already been explored in protein-coding genes-focused studies through single-cell transcriptomics [12, 13].

There is a notable lack of studies addressing the multifaceted dynamics of lncRNA activity across brain regions, cell types, and disease stages. This limitation hampers our understanding of the role of lncRNAs not only in AD pathogenesis but also in adaptive cellular responses for counteracting neurodegeneration. Consequently, this study aimed to characterize lncRNA dynamics in silico across the multifactorial landscape of AD progression within the human cerebral cortex at the single-cell resolution level.

## Methods

### Transcriptomic Data Curation and Selection

Single-nucleus transcriptomic datasets were obtained from the NCBI Gene Expression Omnibus (GEO) using the search terms “scRNA”, “AD”, and “human brain”. Three postmortem cortical regions from male human donors were analyzed: the prefrontal cortex (PFC), the superior frontal gyrus (SFG), and the entorhinal cortex (EC). The SFG dataset (GSE147528) included ten individuals (Braak 0, *n* = 3; Braak II, *n* = 4; Braak V–VI, *n* = 3). The EC dataset was derived from the same donors [14]. The PFC dataset (GSE157827) was restricted to male individuals because female samples were unevenly distributed across Braak stages, which could introduce sex-related bias. Samples at Braak stage IV were excluded to ensure comparability between both datasets, resulting in a final set of twelve individuals (Braak II, *n* = 6; Braak V–VI, *n* = 6) (Supplementary Table S1) [15]. Alignment and gene annotation were made using GRCh38 reference genome, through different versions of CellRanger (v3.0.1 and 2.1, respectively), as explained by the authors of the data-generating papers. Raw count matrices were downloaded in Matrix Market (.mtx) and Hierarchical Data Format (.h5) files, respectively.

### snRNA-seq Data Processing and Quality Control

The collected data was processed in R v4.5 [16] using the Seurat v5.0.0 package [17]. A Seurat object was generated for each sample using the *CreateSeuratObject* function (parameters: ≥ 3 cells and ≥ 200 genes detected per cell) and annotated with the corresponding metadata. Individual objects were merged into a single Seurat object using the *merge* function. No additional dataset integration algorithm was applied at single-cell level data, as analyses were independently conducted for each brain region to preserve biologically relevant transcriptional variation. Quality control measurements were performed with the *PercentageFeatureSet* function to compute the proportion of mitochondrial transcripts, excluding cells with fewer than 200 or more than 2,500 detected total genes or with > 20% mitochondrial content. Data normalization and variance stabilization were conducted using *SCTransform*, which models sequencing depth to correct for differences in total RNA counts across cells while regressing out the effect of mitochondrial transcript proportion. Dimensionality reduction was performed using *RunPCA*, followed by *RunUMAP* on the top 20 principal components. A shared nearest neighbor (SNN) graph was constructed using *FindNeighbors*, and clustering was performed with *FindClusters* across resolutions ranging from 0.0 to 1.0.

### Cell Type Annotation and Quantification

Cell type annotation was performed using ScType [18] with the ScTypeDB database restricted to brain tissue. The scaled expression matrix from the filtered Seurat object was processed by the algorithm to compute similarity scores between clusters and positive/negative marker gene sets. The highest-scoring cell type was assigned to each cluster, and the resulting annotations were integrated into the Seurat metadata. Absolute variation in cell number was evaluated by calculating the number of cells per cell type, sample, region, and condition. From these counts, the mean, standard error of the mean (SEM), and 95% confidence intervals (CI95%) were computed.

### Pseudobulk-based Intercellular Gene Expression Profiles Comparison

Single-cell data were first aggregated into pseudobulk profiles using the *AggregateExpression* function from the Seurat package v5.0.0 [17]. Gene expression was aggregated per sample, AD stage, study and cell type. This strategy reduces cell-level noise and mitigates pseudoreplication by treating each biological group as a single unit. Only genes with counts ≥ 10 in at least three pseudobulk samples were retained. A DESeqDataSet was built via the *DESeqDataSetFromMatrix* function from DESeq2 [19]. Variance-stabilizing transformation was performed, which normalizes count data while accounting for mean–variance dependence, producing homoscedastic expression values suitable for downstream multivariate analyses. To correct for study-specific batch effects, the *ComBat* function from the sva package [20] was applied, incorporating biological covariates (cell type and disease stage) through a design matrix. Principal component analysis (PCA) was conducted on the top variable genes to evaluate global transcriptional structure and the effectiveness of batch correction.

### Graph-based Intercellular Gene Expression Profiles Comparison

For tissue-specific analyses, the complete Seurat object was subdivided by cortical region using the *subset* function. This approach was applied to both the full dataset and a subset containing only the lncRNAs. Each subset was saved in H5Seurat format using *SaveH5Seurat* and subsequently converted to H5AD format, compatible with Scanpy [21], using the *Convert* function. Cell type connectivity analyses were performed using the Partition-based Graph Abstraction (PAGA) algorithm [22] implemented in Scanpy. For each H5AD object, cell subsets corresponding to each disease stage were created, and PAGA graphs were computed to extract connectivity weights between cell types. Changes in these intra-tissue connectivity weights were examined across AD stages. Principal component analysis (PCA) was performed on the connectivity matrices to assess intra-tissue expression variation.

### Differential Expression Analysis

From the previously filtered and preprocessed Seurat object, SCT-normalized counts were extracted and prepared via the *PrepSCTFindMarkers* function [17], which ensures appropriate variance modeling for downstream testing. Cells were stratified into biologically meaningful subsets by concatenating cell type and tissue into a composite grouping variable, and independent Seurat objects were generated for each combination to enable context-specific comparisons. Within each subset, differential expression analyses were performed between AD stages using the *FindMarkers* function, employing the hurdle model implemented in the MAST method [23], which is specifically designed for zero-inflated single-cell data. Donor identity was incorporated as a latent variable, thereby accounting for inter-individual variability and reducing pseudoreplication effects at the single-cell level [24]. Pairwise contrasts were dynamically defined based on the available disease stages within each subset. To control for multiple hypothesis testing, raw p-values were adjusted using the Benjamini–Hochberg false discovery rate (FDR) procedure [25]. Gene annotation was then performed using the Isoformic library [26] and the biomaRt interface to Ensembl [27]. Differentially expressed genes (DEG) were defined based only on statistical significance (FDR < 0.05).

### Cross-study Validation of Differential Expression Signatures

To assess the robustness and reproducibility of our findings, we conducted a systematic cross-study comparison using differential expression results reported in previously published studies. A comprehensive literature search was conducted in PubMed to identify studies with comparable brain regions, cell type annotations, and AD staging. For the prefrontal cortex (PFC), datasets from Morabito et al. 2021 [28], Mathys et al. 2019 [29], and Anderson et al. 2023 [30] were selected. For the entorhinal cortex (EC) and superior frontal gyrus (SFG), we used the datasets from Bartas et al. 2025 [31] and Wang et al. 2024 [32] as references, respectively. Differential expression results were retrieved from the respective supplementary materials of each study. Following data harmonization, raw p-values were uniformly adjusted using the Benjamini–Hochberg method, and DEGs were defined using a significance threshold of FDR < 0.05. To ensure comparability across studies, only DEGs corresponding to advanced versus control disease stages (Braak stages V–VI vs 0) were considered. For EC and SFG, overlap analysis was restricted to pairwise comparisons between our reanalyzed dataset and the corresponding matched study. Overlap was defined as the intersection of significant DEGs between datasets, while concordance was quantified as the proportion of overlapping genes exhibiting consistent directionality of expression change. For the PFC, overlap and concordance were computed across all pairwise study comparisons. Summary statistics, including the mean and standard deviation of overlap and concordance metrics, were calculated for each cell type to provide an aggregate measure of cross-study reproducibility.

### Genomic Regions Tracking

Genomic annotation dictionaries were built using *TxDb* from the GenomicFeatures package [33] to extract genomic coordinates. Transcript-to-gene mappings were established with *make_tx_to_gene* from the Isoformic package [26]. For each lncRNA, ± 1 Mb genomic windows were defined as GRanges objects to identify potential *cis* targets within a functionally relevant distance. The *findOverlaps* function was used to detect genes located within these expanded regions, excluding self-interactions and retaining only potential targets. Overlapping genes were cross-referenced with biotype dictionaries, retaining protein-coding targets only.

### lncRNA-neighbor mRNAs Expression Correlation

For each cell type–tissue combination in the Seurat object, gene expression counts were aggregated at the sample and condition levels using the *AggregateExpression* function, generating pseudobulk expression matrices distinct from those used in the “Pseudobulk-based Intercellular Gene Expression Profiles Comparison” subsection. These were converted from raw counts to TPM using a custom *counts_to_tpm* function to enable relative expression comparisons. For each lncRNA–target pair, Spearman rank correlations were computed using the *rcorr* function from the Hmisc package [34]. Resulting p-values were adjusted for multiple testing using the Benjamini–Hochberg procedure, and only statistically significant correlations (FDR < 0.05) were retained for downstream analyses. No additional filtering based on correlation magnitude was applied, as all significant pairs exhibited an absolute correlation coefficient (|r|) greater than 0.8.

## Results

### Intercellular Expression Reveals Convergent Progression Across Brain Regions

**Fig. 1:**
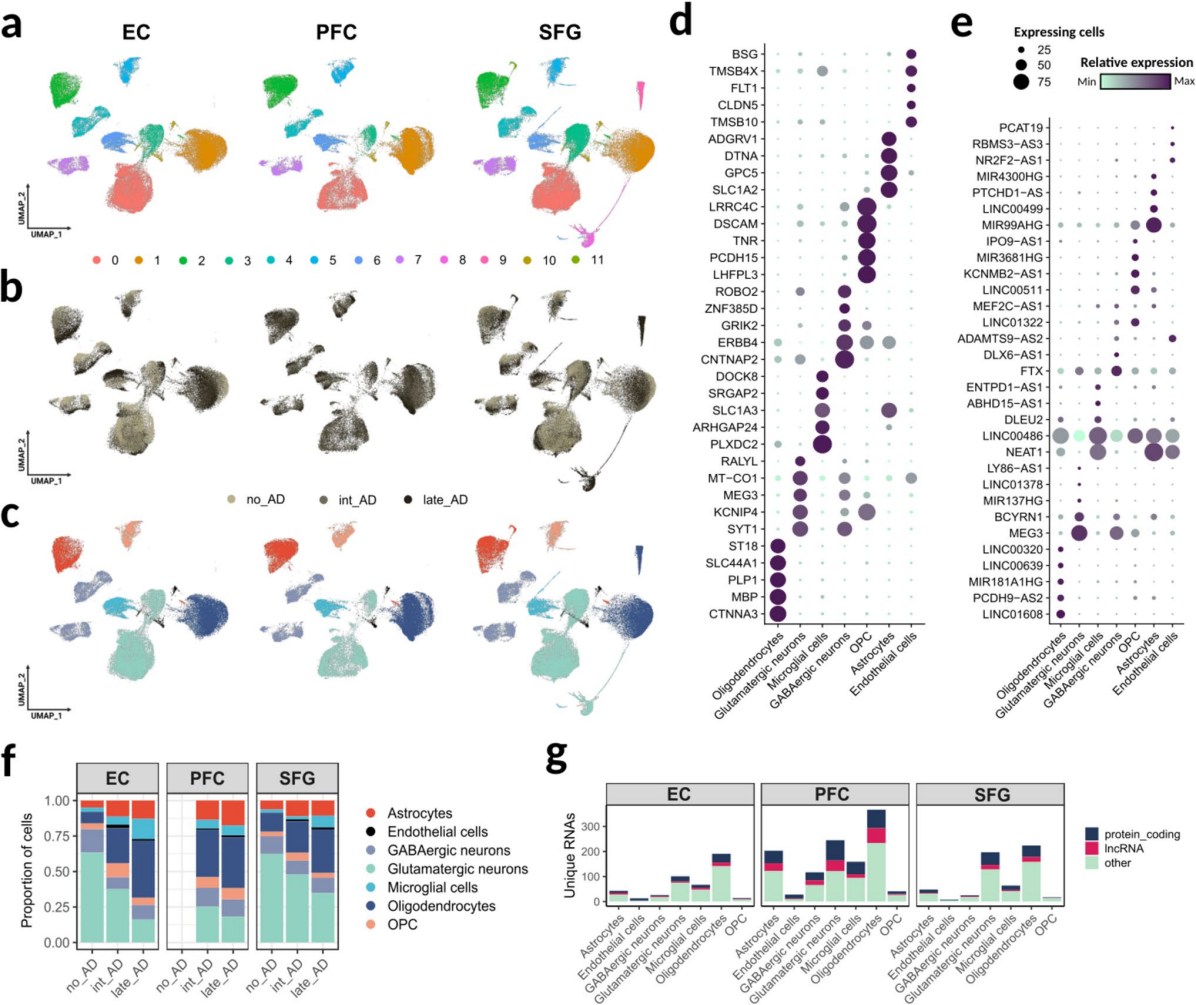
Data characterization. (**A-C**) Dimensionality-reduced representation of all detected cells from both datasets, visualized by (**A**) Louvain clusters, (**B**) disease phase, and (**C**) annotated cell type. (**D**, **E**) Expression levels of cell type-enriched protein-coding genes (**D**) and lncRNAs (**E**). Relative expression levels are shown by the color gradient, whereas the percentage of expressing cells is indicated by dot size. (**F**) Proportion of each cell type within individual brain regions across Alzheimer’s disease (AD) phases. (**G**) Number of genes uniquely detected in each cell type within each brain tissue

After quality control and filtering, the final dataset comprised 239,149 cells derived from three cortical regions: Prefrontal cortex (PFC), superior frontal gyrus (SFG), and entorhinal cortex (EC). In total, 37,341 genes presented non-zero counts in at least one sample, and 32,824 genes were retained after applying quality filters. Among these, 6,778 genes were detected as highly variable features across the datasets. Using the clustering resolution that best reflected cellular structure (resolution = 0.1), we identified 12 distinct clusters (Fig. 1A).

Samples were stratified into three categories based on the donor’s Braak stage: individuals at stage 0 were classified as “no AD”, those at stage II as “intermediate AD”, and those at stages V or VI as “late AD” (Fig. 1B). Most annotated cell clusters were shared across all brain regions; however, clusters 8 and 9 were detected only in the SFG. These clusters were composed of cells derived from a single late-stage AD donor and were not observed in the EC dataset, although both regions were obtained from the same individuals (Supplementary Fig. S1A). Automated cell type annotation using ScType consistently identified seven major cell types across tissues: astrocytes, endothelial cells, excitatory (glutamatergic) neurons, inhibitory (GABAergic) neurons, microglia, oligodendrocytes, and oligodendrocyte precursor cells (OPCs) (Fig. 1C).

Through the whole transcriptome, we identified specific signatures of the cell groups classified as oligodendrocytes (*CTNNA3*, *MBP*, *PLP1*, *SLC44A1* and *ST18*), glutamatergic neurons (*SYT1*, *KCNIP4*, *MEG3*, *MT-CO1* and *RALYL*), microglial cells (*PLXDC2*, *ARHGAP24*, *SLC1A3*, *SRGAP2* and *DOCK8*), GABAergic neurons (*CNTNAP2*, *ERBB4*, *GRIK2*, *ZNF385D* and *ROBO2*), OPCs (*LHFPL3*, *PCDH15*, *TNR*, *DSCAM* and *LRRC4C*), astrocytes (*SLC1A2*, *GPC5*, *DTNA* and *ADGRV1*) and endothelial cells (*TMSB10*, *CLDN5*, *FLT1*, *TMSB4X* and *BSG*) as shown in Fig. 1D. These signatures presented their highest expression levels within the respective cell type and their occurrences were assigned in 50 to 75% of the cells. Those classified as glutamatergic neuron markers were also abundant in inhibitory neurons, while GABAergic neuron markers showed low expression levels in excitatory neurons.

With the exception of *MEG3*, preferentially expressed in glutamatergic neurons (Fig. 1D), no other lncRNA was identified as a major cell type assigner. Because of this, we applied a differential expression test in the lncRNA-only data, which allowed us to identify lncRNAs as significant markers (FDR < 0.05) of oligodendrocytes (*LINC01608*, *PCDH9-AS2*, *MIR181A1HG*, *LINC00639* and *LINC00320*), glutamatergic neurons (*BCYRN1*, *MIR137HG*, *LINC01378* and *LY86-AS1*), microglial cells (*DLEU2*, *ABHD15-AS1* and *ENTPD1-AS1*), GABAergic neurons (*FTX* and *DLX6-AS1*), OPC (*LINC00511*, *KCNMB2-AS1*, *MIR3681HG* and *IPO9-AS1*), astrocytes (*MIR99AHG*, *LINC00499*, *PTCHD1-AS* and *MIR4300HG*) and endothelial cells (*NR2F2-AS1*, *RBMS3-AS3* and *PCAT19*) as illustrated in Fig. 1E.

Both *NEAT1*, *LINC00486*, *ADAMTS9-AS2*, *LINC01322*, and *MEF2C-AS1* presented close expression levels in multiple cell types (Fig. 1E). Notably, the amount of expressing cells was generally higher when considering protein-coding genes as markers (Fig. 1D) when compared to lncRNAs (Fig. 1E). *MEG3*, *NEAT1*, and *LINC00486* stood out as abundant lncRNAs, since they were expressed by more than 75% of specific cell communities.

Proportionally, we observed a predominance of neurons, particularly excitatory neurons, in both EC and SFG from individuals without AD (Fig. 1F). However, as disease severity increased, this predominance was progressively reduced and accompanied by a relative expansion of oligodendroglial, astroglial, and microglial populations. Conversely, the PFC exhibited milder compositional shifts across AD stages, primarily involving astrocytic and glutamatergic neuron populations (Fig. 1F).

In all brain tissues, oligodendrocytes presented the greatest amount of unique RNAs, defined as genes exclusively detected in oligodendrocyte clusters (Fig. 1G). They were followed by glutamatergic neurons, microglial cells, and astrocytes. In all cell types, PFC-derived cells exhibited higher numbers of unique transcripts compared to EC or SFG. We also observed that RNAs with unknown or uncharacterized biotype (classified as “others”) were consistently predominant in all cell types. Protein-coding genes were the predominant class of unique transcripts, followed by lncRNAs, which in some PFC-derived cell types (e.g., oligodendrocytes, GABAergic neurons, and astrocytes) nearly equaled their abundance.

Selecting the top 500 highly variable features, principal component analysis (PCA) of pseudobulk data revealed clear cell-type-based clustering (Supplementary Fig. S1B). In the full transcriptome, samples from different brain regions followed a similar trajectory from “no AD” to “late AD,” indicating consistent progression-associated expression patterns across cell types. Notably, although PFC samples clustered with their respective cell types, they were positioned relatively distant from those derived from other regions.

In contrast, restricting the analysis to lncRNAs resulted in a predominantly dataset-driven clustering pattern, clearly separating PFC-derived samples from those of EC and SFG (Supplementary Fig. S1B). Batch correction using ComBat produced only modest changes, with dataset-driven separation largely preserved and the variance explained by PC1 decreasing slightly (from 34 to 30%) in the lncRNA-only subset.

To further assess this effect, we evaluated the impact of progressively restricting the number of highly variable features. Dataset-driven separation increased as fewer features were included, an effect that was particularly pronounced in the lncRNA-only analysis (Supplementary Fig. S1C). Although batch correction performed better in reducing the variance explained by PC1 in this subset, it did not eliminate the observed clustering patterns.

**Fig. 2:**
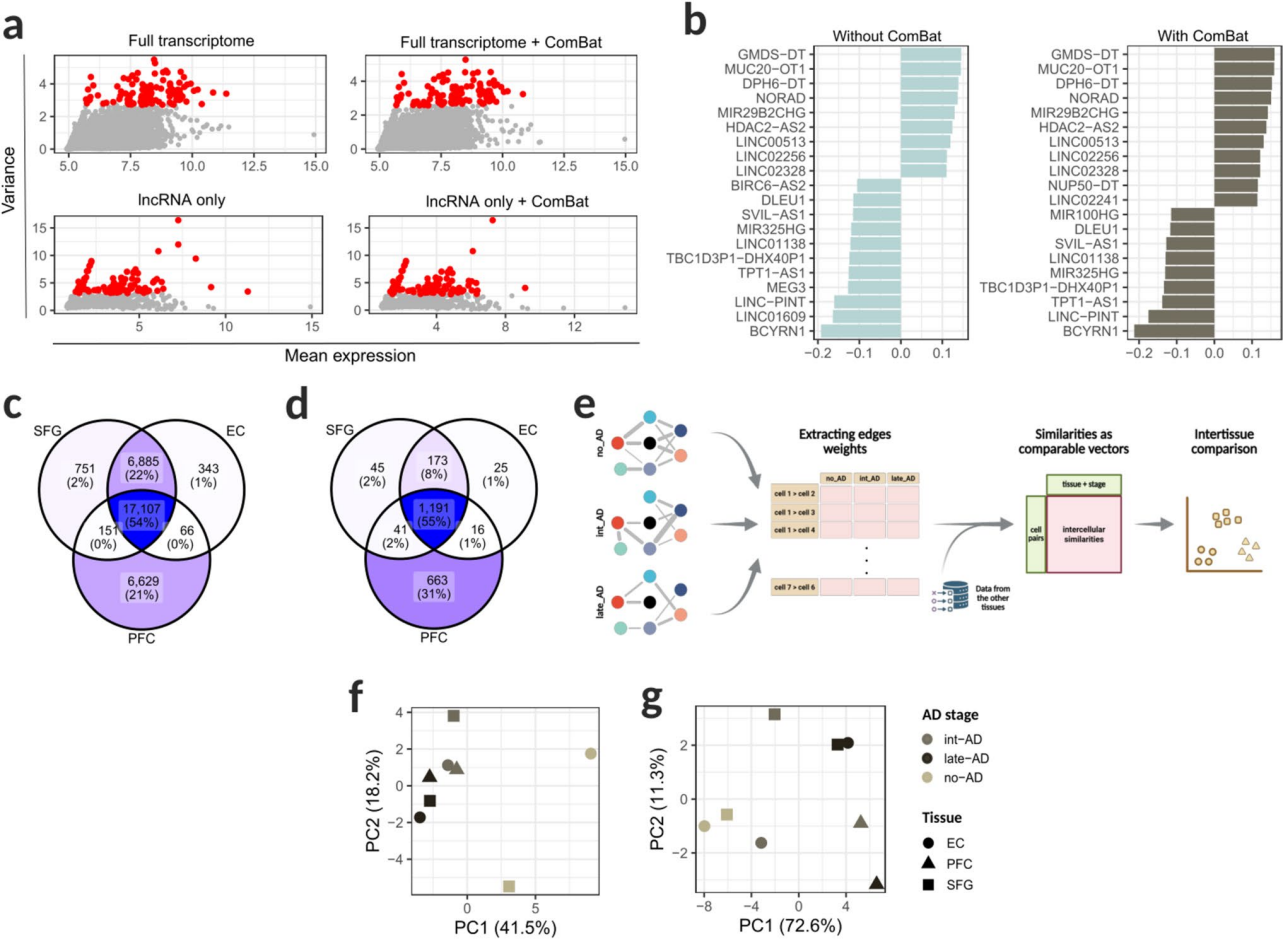
Intertissue comparison. (**A**) Distribution of features according to normalized mean expression (x-axis) and variance across samples (y-axis) in the complete Seurat object and in the lncRNA-only subset, with and without batch correction. Red dots indicate highly variable features (variance > 2.5). (**B**) Top 20 features contributing most to PC1 in the lncRNA-only subset, with and without batch correction. Features are shown on the y-axis and PC1 loadings on the x-axis. (**C**, **D**) Overlap of features identified within each brain tissue for (**C**) the whole transcriptome and (**D**) lncRNA-only data. Darker blue tones indicate higher percentages of overlapping genes. (**E**) Schematic overview of the intertissue comparison workflow. Distances between cell clusters were computed using a graph abstraction–based approach and used as comparable vectors for direct intertissue analysis. (**F**, **G**) Principal component analysis (PCA) of edge weights from PAGA-derived intercellular connectivity networks generated for each tissue–disease stage combined data, based on (**F**) the whole transcriptome and (**G**) lncRNA-only datasets

We next examined the relationship between gene expression level and variance across samples. In the full dataset, variance was broadly distributed among moderately expressed genes (6 to 11 TPM), whereas in the lncRNA-only subset, it was predominantly concentrated in lowly expressed transcripts (2 to 5 TPM) (Fig. 2A). This distribution suggests that the observed clustering is more susceptible to detection-related noise. Consistently, lncRNAs contributing most strongly to dataset separation (PC1 loadings) remained largely unchanged after batch correction (Fig. 2B), indicating that this effect was not fully accounted for by conventional batch adjustment methods.

Together, these findings suggest that the observed dataset-driven clustering is primarily driven by systematic differences in transcript detection between datasets, disproportionately affecting lowly expressed lncRNAs. Although the majority of genes were consistently detected across all three brain regions (54%), a substantial fraction was either uniquely identified in PFC (21%) or shared exclusively between SFG and EC (22%) (Fig. 2C). When the analysis was restricted to lncRNAs (Fig. 2D), a comparable overall overlap was observed (55%); however, the proportion of transcripts uniquely detected in PFC markedly increased (31%), further supporting the contribution of dataset-specific detection biases.

To enable more comparable analyses across brain regions, we next adopted a network-based approach that captures transcriptional relationships within each tissue-disease stage condition. Using partition-based graph abstraction (PAGA), intercellular transcriptomic similarities were quantified as edge weights and subsequently represented as comparable vectors (Fig. 2E). When applied to the full transcriptome, this framework revealed a unified trajectory shared across all brain regions during AD progression (Fig. 2F). In contrast, analysis restricted to lncRNAs recapitulated this pattern for EC and SFG samples.  However, it showed a clear separation of PFC-derived samples (Fig. 2G), consistent with the dataset-specific detection biases described above.

Within all transcriptome-containing PFC, the highest intercellular similarity was found between endothelial and microglial cells (around 20%) at the Braak stage II (Supplementary Fig. S2A). The same was observed in EC, with microglial and endothelial cells sharing a similarity of more than 75% at the Braak stage 0 (Supplementary Fig. S2B). In both cases, AD progression culminated in a drop in similarity to less than 5% at the final stage. No similarity higher than 10% was detected in SFG independently from the cell type (Supplementary Fig. S2C).

PAGA connections generated by only lncRNA-containing data evidenced difficulty in establishing clear transcriptome distinctions between the cell type, with similarities near to 100% in all brain tissues (Supplementary Fig. S2D-F). PFC exhibited most discrete fluctuations again, not presenting any alteration higher than 15% (Supplementary Fig. S2D). On the other hand, connection weights between glial-glial and glial-endothelial cells showed a general decrease in both EC and SFG, ranging from 100% to less than 25% in some cases (Supplementary Fig. S2E and F).

### Cell-Type-Specific lncRNA Dynamics is Shaped By Tissue Context During AD Progression

For differential expression analysis, performed at the single-cell level within each tissue-derived cell type across consecutive AD stages, we applied the Benjamini–Hochberg method for multiple testing correction, as it is less stringent than Seurat’s default Bonferroni approach (Supplementary Fig. S3A). This choice was particularly important for lncRNAs, enabling the retention of a sufficient number (≥ 1) of DEGs for downstream analyses (Supplementary Table S2). We did not impose any fold-change threshold, as our aim was to capture the broadest possible spectrum of lncRNA expression dynamics across brain regions and disease stages (Supplementary Fig. S3B).

**Fig. 3:**
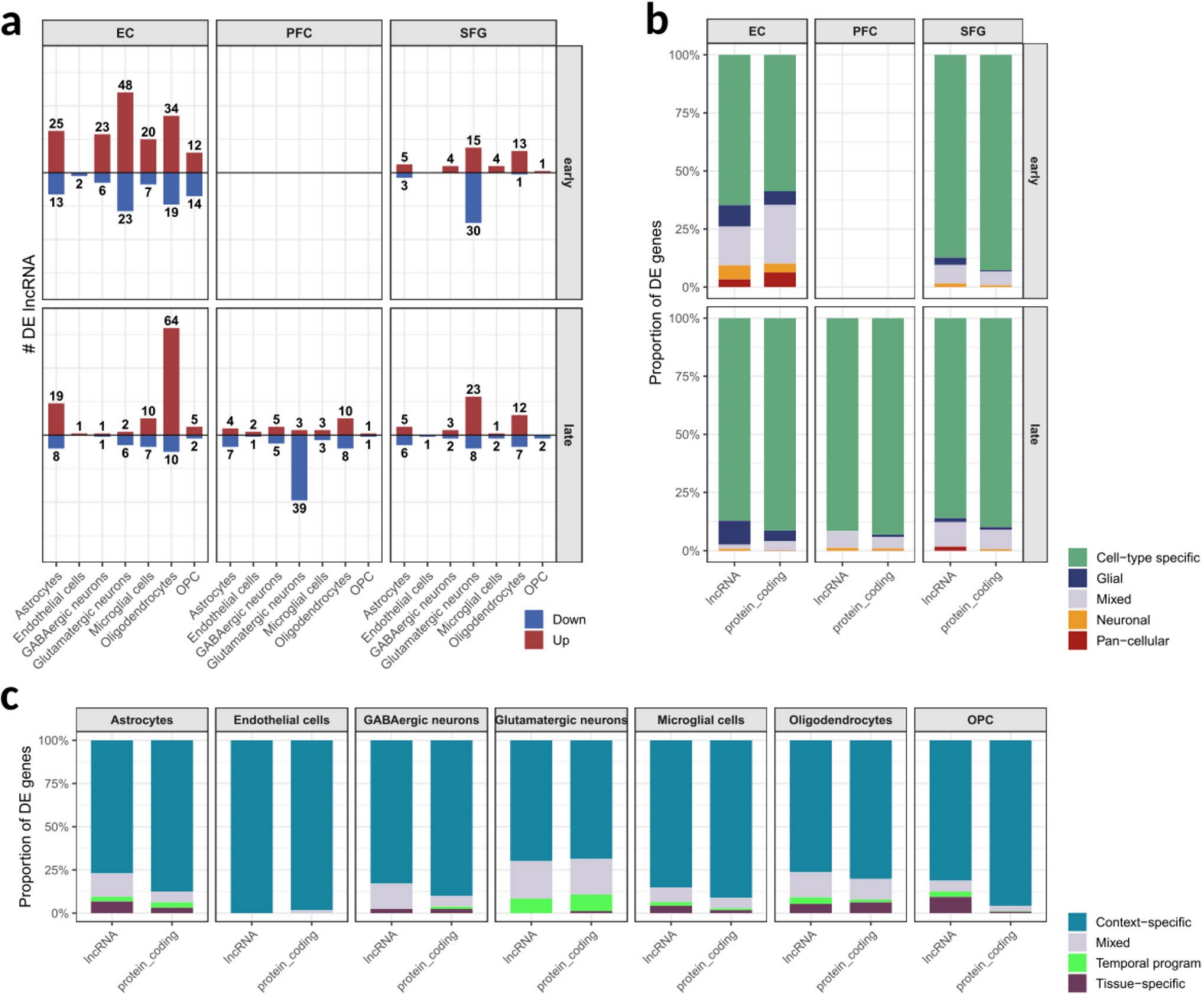
Patterns of differently expressed lncRNAs. (**A**) Number of differentially expressed lncRNAs in each cell type, faceted by brain tissue and AD stage. Upregulated and downregulated genes are distinguished by bar color. (**B**) Cell-type specificity of differentially expressed lncRNAs across tissues and AD stages. Categories: Cell-type specific (altered in only one cell type within a given tissue-stage context); Glial (altered in at least two glial cell types); Neuronal (altered in both excitatory and inhibitory neurons only); Pan-cellular (altered in at least five cell types); and Mixed (patterns not fitting the previous categories). (**C**) Spatiotemporal specificity of differentially expressed lncRNAs across cell types. Categories: Context-specific (altered in a single tissue-stage combination within a given cell type); Temporal program (altered across tissues within a specific AD stage only); Tissue-specific (altered in one tissue across both AD stages); and Mixed (unclassified patterns)

During early AD, glutamatergic neurons from the EC exhibited the highest number of lncRNAs showing upregulated expression (*n* = 48), followed by oligodendrocytes (*n* = 34) and astrocytes (*n* = 25). With the exception of endothelial cells, which showed only two downregulated lncRNAs, all other cell types displayed a predominance of upregulated over downregulated lncRNAs (Fig. 3A). Overall, EC-derived cell types exhibited a greater number of altered lncRNAs compared to those from the SFG, although SFG-derived glutamatergic neurons showed a higher amount of downregulated lncRNAs (*n* = 30).

In late AD, most cell types in EC showed a reduction in the number of differentially expressed lncRNAs compared to the early stage, with the notable exception of oligodendrocytes, which exhibited a marked increase in upregulated lncRNAs (*n* = 64). In SFG, glutamatergic neurons again displayed the highest number of altered lncRNAs, with upregulation predominating (23 upregulated vs. 8 downregulated). This cell type also stood out in the prefrontal cortex (PFC), where a predominance of downregulation was observed (39 downregulated vs. 3 upregulated lncRNAs) (Fig. 3A).

Despite these patterns, lncRNAs consistently represented a small fraction of the total transcriptional changes, accounting for less than 5% of all DEGs across cell types, brain regions, and disease stages (Supplementary Fig. S3C). Nevertheless, both lncRNAs and protein-coding genes exhibited comparable levels of cell-type specificity across all tissue- and stage-specific contexts, defined as genes differentially expressed in only a single cell type within a given condition (Fig. 3B). The same proximity was observed in terms of the spatiotemporal specificity across all analyzed cell types (Fig. 3C). Membership matrices are provided for each cell type for both lncRNAs (Supplementary Table S3) and protein-coding genes (Supplementary Table S4), as well as across all spatiotemporal contexts (Supplementary Tables S5 and S6).

We further compared our differential expression results with previously published studies. Following a comprehensive search aimed at identifying datasets with study designs comparable to ours, three datasets were selected for PFC comparisons [28–30], whereas only one suitable study was identified for EC [31] and SFG [32]. Overlaps between DEGs identified in our analysis and in the reference studies were assessed on a per-cell type basis and defined as replication. Replication rates varied substantially across cell types, even within the same tissue (e.g. from ~ 75% in astrocytes to ~ 20% in GABAergic neurons in EC) while concordance in the direction of expression changes generally exceeded 50% (Supplementary Fig. S4). In PFC, where multiple reference datasets were available, we additionally observed marked inter-study variability, reflected by wide dispersion in replication and concordance estimates across studies.

### Genomic Context–Based Inference of lncRNA Functional Involvement

Differentially expressed lncRNAs were distributed across both gene-rich and gene-poor genomic regions, as defined by the number of neighboring mRNAs within a ± 1 Mb window (data not shown). To further explore their potential functional roles, we assessed the correlation between altered lncRNAs and nearby protein-coding genes, aiming to identify coordinated expression patterns, infer putative *cis*-regulatory relationships, and contextualize these interactions within their genomic environment (Supplementary Tables S7 and S8). In addition, publicly available Micro-C data from the UCSC Genome Browser [35] were incorporated to evaluate whether these lncRNA-mRNA pairs are also spatially associated within three-dimensional chromatin structures.

**Fig. 4:**
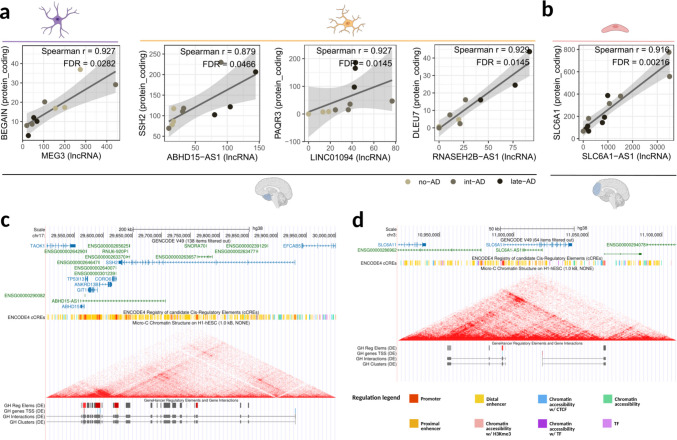
Antisense lncRNAs in non-neuronal cell types. (**A**, **B**) Pseudobulk expression-based correlations between differentially expressed lncRNAs and proximal (± 1 Mb) differentially expressed protein-coding genes in (**A**) EC-derived astrocytes and microglia (left to right), and (B) PFC-derived endothelial cells. Each dot represents one sample, colored accordingly to its respective AD stage. (**C**, **D**) Genomic loci of (**C**) *ABHD15-AS1* and (**D**) *SLC6A1-AS1*. Tracks were obtained from the UCSC Genome Browser and include GENCODE v49, ENCODE cCREs, GeneHancer, and Hi-C/Micro-C data. (http://genome.ucsc.edu)

In astrocytes from EC, Maternally Expressed Gene 3 (*MEG3*) showed strong positive correlation with Brain-Enriched Guanylate Kinase-Associated Protein (*BEGAIN*) (r = 0.927), with both genes downregulated during early AD (*MEG3* log2FC =  − 0.95; *BEGAIN* log2FC =  − 1.18). In microglia from the same tissue, three positively correlated lncRNA-mRNA pairs were identified, all showing coordinated upregulation during disease progression: ABHD15 Antisense RNA 1 (*ABHD15-AS1*) with Slingshot Protein Phosphatase 2 (*SSH2*), Long Intergenic Non-Protein Coding RNA 1094 (*LINC01094*) with Progestin and AdipoQ Receptor Family Member 3 (*PAQR3*), and RNASEH2B Antisense RNA 1 (*RNASEH2B-AS1*) with Deleted in Lymphocytic Leukemia 7 (*DLEU7*) (Fig. 4A).

In the endothelial cells from PFC, expression of Solute Carrier Family 6 Member 1 (*SLC6A1*) was positively correlated with its Antisense RNA 1 (*SLC6A1-AS1*) (Fig. 4B). They were simultaneously downregulated from Braak stage 2 to 6. Within both regions, antisense lncRNAs exhibited coordinated expression with their sense counterparts. However, while in PFC the correlated pair involved a protein-coding gene and its directly overlapping antisense lncRNA, in EC a similar relationship was observed between genes with distinct annotations (e.g., *SSH2* and *ABHD15-AS1*) that nevertheless share antisense genomic positioning (Fig. 4C, D). In both contexts, these gene pairs were located within the same chromatin interaction domains.

**Fig. 5:**
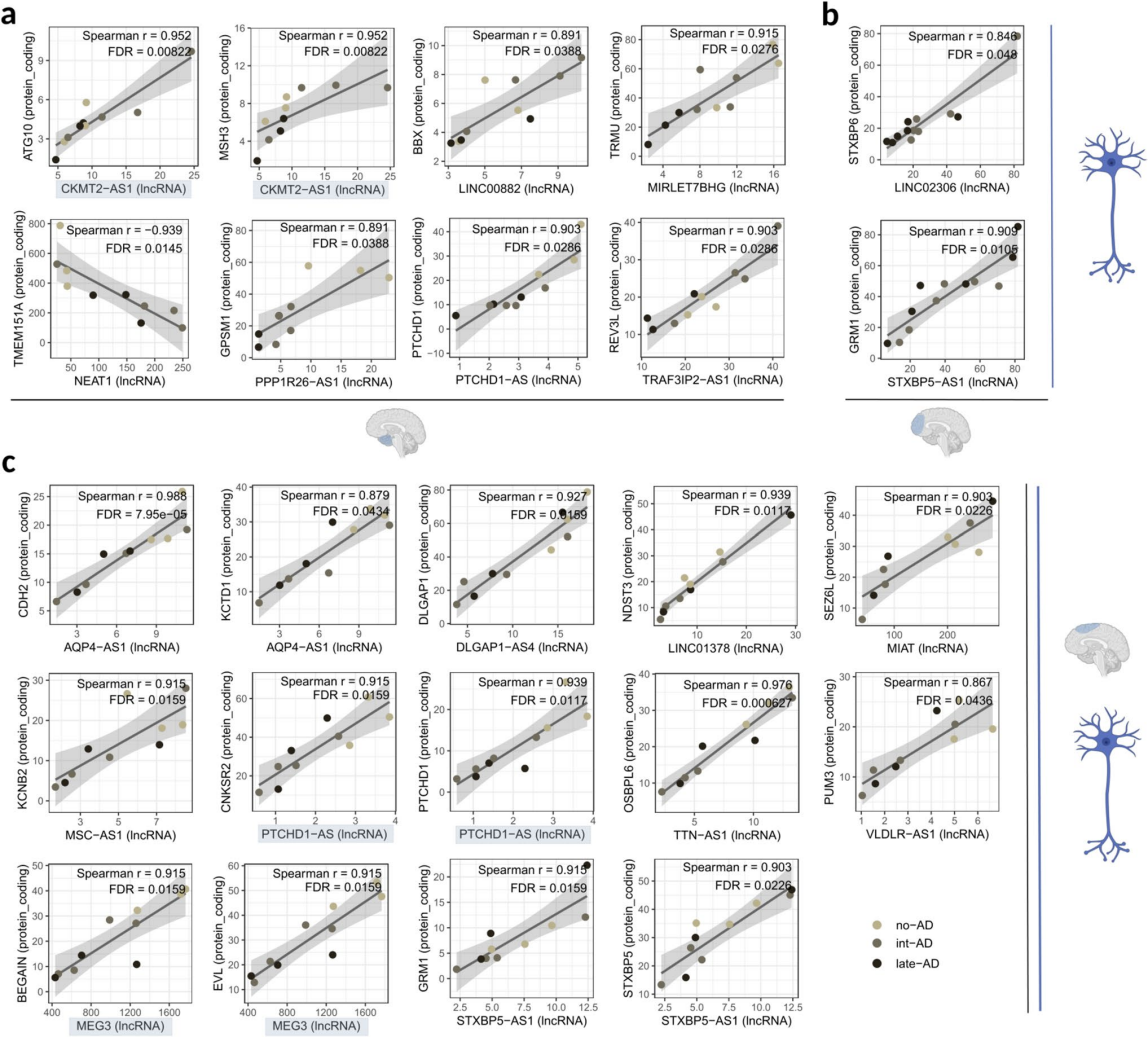
Single lncRNAs associated with multiple protein-coding genes in excitatory neurons. (**A-C**) Pseudobulk expression-based correlations between differentially expressed lncRNAs and proximal (± 1 Mb) differentially expressed protein-coding genes in glutamatergic neurons derived from (**A**) entorhinal cortex, (**B**) prefrontal cortex, and (**C**) superior frontal gyrus. Each dot represents one sample, colored accordingly to its respective AD stage

No significant lncRNA-mRNA correlations were detected in GABAergic neurons across the analyzed tissues. In contrast, glutamatergic neurons consistently exhibited such associations in all three brain regions (Fig. 5). In EC-derived glutamatergic neurons, eight correlated pairs were identified, including two involving the same lncRNA. Notably, CKMT2 Antisense RNA 1 (*CKMT2-AS1*) showed strong positive correlations with both Autophagy Related 10 (*ATG10*) and MutS Homolog 3 (*MSH3*) (r = 0.952 for both), with all three genes upregulated during early AD phase (*CKMT2-AS1* log2FC = 0.91; *ATG10* log2FC = 0.25; *MSH3* log2FC = 0.52) (Fig. 5A).

In PFC-derived glutamatergic neurons, only two correlated pairs were detected: Long Intergenic Non-Protein Coding RNA 2306 (*LINC02306*) with Syntaxin Binding Protein 6 (*STXBP6*), and STXBP5 Antisense RNA 1 (*STXBP5-AS1*) with Glutamate Metabotropic Receptor 1 (*GRM1*) (Fig. 5B). In contrast, fourteen correlated pairs were identified in SFG-derived glutamatergic neurons, including recurrent associations involving PTCHD1 Antisense RNA (*PTCHD1-AS*) and *MEG3* (Fig. 5C). Analysis of chromatin organization revealed that the mRNAs correlated with the same lncRNAs are distributed across both shared and adjacent chromatin interaction domains (Supplementary Fig. S5).

**Fig. 6:**
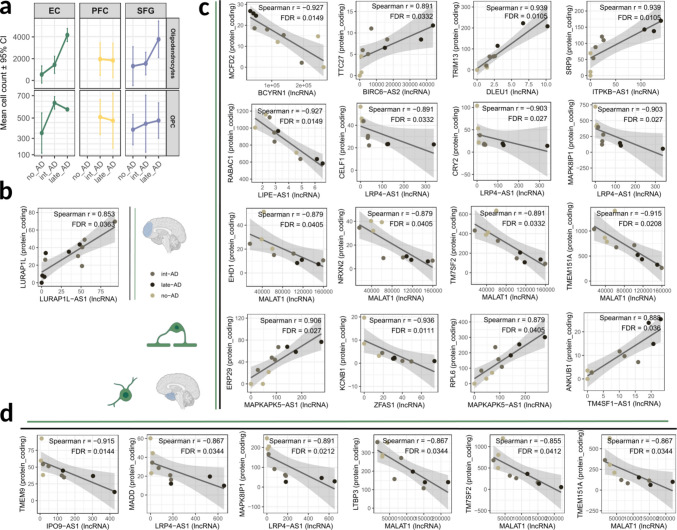
Oligodendroglial-specific alterations during AD progression. (**A**) Abundance of oligodendrocytes and OPCs across brain regions and AD stages. Each dot represents the mean number of cells per sample; error bars show 95% confidence intervals. (**B-D**) Pseudobulk expression-based correlations between differentially expressed lncRNAs and proximal (± 1 Mb) differentially expressed protein-coding genes in oligodendrocytes from (**B**) prefrontal cortex and (**C**) entorhinal cortex, and in OPCs from (**D**) entorhinal cortex. Each dot represents one sample, colored accordingly to its respective AD stage

Despite proportional changes in cellular composition across AD stages (Fig. 1F), relatively few cell populations exhibited significant alterations in abundance (Supplementary Fig. S6). No significant changes were detected in PFC, whereas microglial expansion was observed in SFG during late AD. The EC displayed the most pronounced compositional shifts, including expansion of astrocytic and microglial populations, as well as dynamic changes in the oligodendroglial lineage. Notably, OPCs increased during early AD, followed by an expansion of mature oligodendrocytes (Fig. 6A).

Region-specific lncRNA-mRNA correlations were also evident in oligodendrocytes. In PFC, only one significant pair was identified, involving the coordinated downregulation of Leucine Rich Adaptor Protein 1 Like (*LURAP1L*; log2FC =  − 1) and its Antisense RNA 1 (*LURAP1L-AS1*; log2FC =  − 2) during late AD phase (r = 0.85) (Fig. 6B). In contrast, sixteen correlated pairs were identified in EC-derived oligodendrocytes (Fig. 6C), including multiple associations involving LRP4 Antisense RNA 1 (*LRP4-AS1*; three pairs) and Metastasis Associated Lung Adenocarcinoma Transcript 1 (*MALAT1*; four pairs). These correlations were exclusively negative, linking upregulation of the lncRNAs with downregulation of their associated mRNAs.

These lncRNAs also stood out in OPCs from the same region, being involved in five out of six identified correlation pairs (Fig. 6D). The expression of *LRP4-AS1* was negatively correlated with Mitogen-Activated Protein Kinase 8 Interacting Protein 1 (*MAPK8IP1*) expression in both OPCs and mature oligodendrocytes (r =  − 0.9 for both), while MALAT1 showed overlapping associations with Transmembrane 7 Superfamily Member 2 (*TM7SF2*) and Transmembrane Protein 151 A (*TMEM151A*). Given that *MALAT1* exhibited the highest number of correlated targets in EC-derived oligodendroglial populations, its genomic locus was further examined, revealing an extended region of potential regulatory influence (Supplementary Fig. S7).

## Discussion

As a highly complex pathology, AD is accompanied by heterogeneous cellular [36, 37], biochemical [38, 39] and clinical alterations [40, 41] based on the analyzed tissue or disease stage. Single-cell transcriptomics (e.g. scRNA-seq or snRNA-seq) has been successfully applied in order to increase our comprehension about these heterogeneous cell populations during AD progression [12, 14, 15]. The application of these techniques to investigate lncRNA roles is not a novelty [42, 43], and has proven useful in detecting genes with restricted abundance, as reviewed by Mattick et al. [44] and Grammatikakis et al. [45]. Nevertheless, here we provide a broader panel of lncRNAs of interest in AD, congregating multiple studies into a single analysis and using an unbiased clustering and correlation approach.

The adoption of an automatic cell type annotator method aimed at providing an unbiased perspective of the data composition [46]. ScType is a widely used tool in single-cell transcriptomics due to its high annotation accuracy (> 98%), even in complex tissues such as the human brain [18, 47]. Its outstanding performance derives from the comprehensive marker genes-based database supporting the algorithm, but also from the score strategy employed to classify both positive and negative markers, improving robustness [18]. Using this approach, we identified the same major cellular populations reported in the study describing the GSE147528 dataset [14]. In addition, we further stratified the oligodendroglial cell cluster into mature oligodendrocytes and OPCs, which represents a refinement compared to the classification reported in the original study that generated the GSE157827 dataset [15].

Most of the cell-type marker genes identified in this study were consistent with those reported in the original data-generating studies, including *MBP*, *ST18*, *PLP1*, and *CTNNA3* for oligodendrocytes; *CLDN5* and *FLT1* for endothelial cells; *SLC1A2*, *ADGRV1*, and *GPC5* for astrocytes; *RALYL* and *KCNIP4* for glutamatergic neurons; and *DOCK8*, *ARHGAP24*, and *PLXDC2* for microglial cells [14, 15]. Additional overlaps were observed with cell-type markers previously reported in transcriptomic studies based on isolated brain cell populations, including *SYT1* and *ROBO2* for neurons [48, 49]; *PCDH15* and *LHFPL3* for OPCs [49]; *PLP1* for oligodendrocytes [48]; and *CLDN5*, *BSG*, and *FLT1* for endothelial cells [48, 49]. More abundant lncRNAs were also assigned as cell type markers. Noticeably, both *MEG3* and Brain Cytoplasmic RNA 1 (*BCYRN1* or *BC200*) are known for their prominent effects on neuronal cells [50, 51]. *ENTPD1-AS1* was also identified as a microglial marker, as well as its sense protein-coding gene [52].

Part of our analysis was also designed to mitigate batch effects in the single cell-level data using an alternative strategy. To accommodate the inherent variability between studies, we employed a graph abstraction approach based on partition-based graph abstraction (PAGA), in which each AD phase-brain tissue combination was treated as an independent context [22]. This represents an extension of the original algorithmic framework and operates conceptually similarly to distance-neighborhood approaches [53], effectively normalizing intercellular similarities through condition-specific cluster distances. This strategy allowed us to preserve biologically meaningful variation across Braak stages while reducing study-specific effects.

The proposed methodology worked well on all transcriptome-containing data, but not on only lncRNA-containing data. Since the obtained clustering profile reflects the isolation of PFC from the other tissues (Fig. 2G), we consider that this occurred due to the greater impact that methodological specificities between the two studies could have on the identification and quantification of lncRNA transcripts. Once they are generally expressed in lower abundances when compared to protein-coding genes, dissecting their entire composition within each cell type can be especially challenging to single-cell transcriptomics technologies due to the sequencing depth limitation [54, 55].

High-throughput transcriptomic pipelines pose particular challenges for lncRNA analysis [56, 57]. Stringent quantification limits and statistical thresholds [58] often reduce the detection of subtle yet biologically relevant changes in lncRNA expression, thereby underestimating their functional contributions. Validation efforts are similarly constrained by these technical limitations, contributing to limited overlap across studies [59]. Despite these challenges, we identified a robust panel of dysregulated lncRNAs across multiple tissues and cell types during AD progression. By integrating expression correlation analyses with proximal genes (within 1 Mb) and Micro-C chromatin interaction data, we further inferred potential functional roles for these lncRNAs.

Antisense-mediated regulation represents one of the most prevalent mechanisms by which *cis*-acting lncRNAs exert their functions [10]. These genes can act as either enhancers or repressors of their associated genes and are frequently transcribed from enhancer-associated loci [44]. For instance, the coordinated upregulation of *ABHD15-AS1* and *SSH2* in EC-derived microglia suggests potential *cis*-regulatory interactions or shared regulatory control within the same genomic locus. This observation is particularly relevant given that the SSH protein family plays a well-established role in regulating cytoskeletal dynamics in microglia through cofilin modulation [60–62].

Similarly, *SLC6A1-AS1* and *PTCHD1-AS* emerge as strong candidates for further investigation due to their correlation with their sense protein-coding counterparts. *SLC6A1* encodes a GABA transporter essential for neurotransmitter uptake [63, 64], whereas PTCHD1 functions as a postsynaptic scaffold protein critical for excitatory synapse integrity [65, 66]. Our findings suggest that their antisense transcripts may act as positive regulators of gene expression or reflect cell state-specific regulatory programmes in response to AD-associated stimuli.

Broader genomic effects were also evident in loci where lncRNAs exhibited coordinated correlations with multiple neighboring mRNAs. Notably, loci containing *MEG3* and *CKMT2-AS1* displayed patterns consistent with higher-order chromatin organization, suggesting their involvement in disease-associated cellular reprogramming [67, 68]. These observations raise the possibility that such lncRNAs may not only respond to but also actively contribute to epigenetic remodeling processes [69, 70]. In this context, the functional identity of nearby protein-coding genes is particularly informative. For example, BEGAIN, a regulator of synaptic transmission and plasticity, is implicated in cognitive processes such as learning and memory [71].

Accumulating evidence indicates that oligodendrocyte lineage cells are highly susceptible to aging and AD, contributing to early myelin disruption and impaired remyelination [72, 73]. Recent studies have demonstrated that OPCs exhibit reduced regenerative capacity and undergo senescence-associated changes in the aging brain [74]. These changes impair differentiation, potentially leading to the accumulation of dysfunctional progenitors despite increased proliferative activity [75]. Consequently, defective differentiation may result in impaired myelination, even in the presence of increased oligodendrocyte abundance [76–78].

Our findings provide additional insight into the potential involvement of lncRNAs in this oligodendroglial context. *MALAT1*, for instance, is widely expressed across oligodendrocyte lineage cells, including both progenitor and mature states [79], suggesting a potential role in regulating transcriptional programmes associated with oligodendroglial function. Although its direct contribution to oligodendrocyte differentiation remains unclear, *MALAT1* is known to modulate chromatin architecture and gene expression across diverse cellular systems [80, 81], supporting its functional versatility.

Taken together, the present study provides a comprehensive overview of lncRNA expression patterns across multiple brain regions and cell types during AD progression. However, our conclusions are primarily based on computational inference, and therefore do not establish causality. The complexity of lncRNA biology, combined with technical limitations inherent to single-cell transcriptomics, reinforces the need for functional validation using experimental models. It will be essential to determine whether the identified lncRNAs actively drive, modulate, or simply reflect the observed transcriptional and cellular changes. Rather than providing definitive mechanistic conclusions, this work aims to serve as a rich resource and hypothesis-generating framework, prioritising lncRNAs and genomic loci for targeted investigation.

## Conclusion

The present study provides a cross-regional, single-cell view of lncRNA dynamics during AD progression, revealing convergent cellular trajectories alongside marked tissue-specific transcriptional signatures. Despite technical constraints inherent to lncRNA detection, we identify reproducible, cell-type-specific lncRNA alterations and highlight coordinated lncRNA-mRNA relationships within defined genomic and chromatin contexts. These findings point to potential roles for lncRNAs in key processes underlying AD pathology. While our results are primarily hypothesis-generating, they establish a prioritized framework of candidate lncRNAs and regulatory loci for future functional studies aimed at elucidating their mechanistic contributions to neurodegeneration.

## Contribution

Conceptualization: AC, IM, GRF, EMR and HU; Writing – review & editing: AC, IM, LS, GJ, FTV, GRF, YT, EMR and HU; Writing – original draft: AC; Project administration: HU; Investigation: AC; Methodology: AC and LS; Funding acquisition: HU; Supervision: HU.

## Supplementary Information

Below is the link to the electronic supplementary material.

ESM 1 Batch effect correction. (A) Dimensionality-reduced representation of all detected cells from both datasets, visualized by sample ID. (B) Pseudobulk PCA of all cell type-brain region-AD stage combinations using the full transcriptome and lncRNA-only datasets, with and without batch correction. Cell types are indicated by color, brain region by point shape, and AD stage by border color. (C) Variance explained by PC1 across reducing numbers of top variable features. Solid lines represent uncorrected data, dashed lines represent batch-corrected data; colors allow to distinguish between full transcriptome and lncRNA-only datasets (PNG 1.34 MB)

High resolution image (TIFF 778 KB)

ESM 2 Variation in intercellular transcriptomic similarity. (A-C) Full transcriptome-derived PAGA-extracted intercellular connection variations across AD stages within prefrontal cortex, entorhinal cortex and superior frontal gyrus, respectively. (D-F) lncRNA-only PAGA-extracted intercellular connection variations across AD stages within prefrontal cortex, entorhinal cortex and superior frontal gyrus, respectively (PNG 1.62 MB)

High resolution image (TIFF 693 KB)

ESM 3 Thresholds for differential expression of lncRNAs. (A) Number of significant DEGs per cell type and region according to different p-value adjustment methods. (B) Number of differentially expressed lncRNAs across log2 fold-change thresholds. (C) Proportion of the altered transcriptome represented by lncRNAs across cell types, faceted by tissue. Bar colors indicate AD stage; the y-axis also reflects absolute counts of dysregulated lncRNAs (PNG 759 KB)

High resolution image (TIFF 416 KB)

ESM 4 Cross-study comparison of differential expression results. For each cell type within a given tissue, replication was defined as the number of overlapping significant DEGs between pairwise comparisons across studies. Concordance was defined as the proportion of replicated DEGs showing the same direction of expression change (PNG 358 KB)

High resolution image (TIFF 170 KB)

ESM 5 Genomic regions with multiple correlated genes. (A-C) Genomic loci of (A) MEG3, (B) CKMT2-AS1, and (C) PTCHD1-AS. Tracks were obtained from the UCSC Genome Browser (GENCODE v49, ENCODE cCREs, GeneHancer, Hi-C/Micro-C). (http://genome.ucsc.edu). Highlighted genes indicate lncRNAs and protein-coding genes with correlated expression (PNG 2.13 MB)

High resolution image (TIFF 1.87 MB)

ESM 6 Cell-type abundance across AD progression. Abundance of astrocytes, endothelial cells, GABAergic neurons, glutamatergic neurons and microglia across brain regions and AD stages. Each dot represents the mean number of cells per sample; error bars show 95% confidence intervals (PNG 334 KB)

High resolution image (TIFF 181 KB)

ESM 7 Genomic region of MALAT1 locus. Genomic map obtained from UCSC Genome Browser and the displayed tracks are: GENCODE V49, ENCODE cCREs, GeneHancer and Hi-C and Micro-C (http://genome.ucsc.edu). MALAT1 and protein-coding genes with correlated expression are highlighted (PNG 1.71 MB)

High resolution image (TIFF 1.59 MB)

ESM 8 (XLSX 21.5 MB)

ESM 9 (XLSX 2.16 MB)

ESM 10 (XLSX 342 KB)

## Data Availability

Transcriptome data is available at Gene Omnibus (GEO) repository from NCBI, under their original IDs: GSE157827 (PFC data) and GSE147528 (EC and SFG data). Data generated from the analysis presented here is all available as supplementary material. The entire code to reproduce these analyses is available at: [https://github.com/Allan-bqi/lncRNAs-AD].

## References

[1] Li X, Feng X, Sun X, Hou N, Han F, Liu Y (2022) Global, regional, and national burden of Alzheimer’s disease and other dementias, 1990-2019. Front Aging Neurosci 14:937486. 10.3389/fnagi.2022.937486 36299608 10.3389/fnagi.2022.937486 PMC9588915

[2] Hinz FI, Geschwind DH (2017) Molecular genetics of neurodegenerative dementias. Cold Spring Harb Perspect Biol. 10.1101/cshperspect.a023705 27940516 10.1101/cshperspect.a023705 PMC5378052

[3] Behl C (2024) In 2024, the amyloid-cascade-hypothesis still remains a working hypothesis, no less but certainly no more. Front Aging Neurosci 16:1459224. 10.3389/fnagi.2024.1459224 39295642 10.3389/fnagi.2024.1459224 PMC11408168

[4] Scheltens P, De Strooper B, Kivipelto M, Holstege H, Chételat G, Teunissen CE et al (2021) Alzheimer’s disease. Lancet 397:1577–1590. 10.1016/s0140-6736(20)32205-4 33667416 10.1016/S0140-6736(20)32205-4 PMC8354300

[5] Braak H, Braak E (1991) Neuropathological stageing of Alzheimer-related changes. Acta Neuropathol 82:239–259. 10.1007/bf00308809 1759558 10.1007/BF00308809

[6] Therriault J, Pascoal TA, Lussier FZ, Tissot C, Chamoun M, Bezgin G et al (2022) Biomarker modeling of Alzheimer’s disease using PET-based Braak staging. Nat Aging 2:526–535. 10.1038/s43587-022-00204-0 37118445 10.1038/s43587-022-00204-0 PMC10154209

[7] Lauretti E, Dabrowski K, Praticò D (2021) The neurobiology of non-coding RNAs and Alzheimer’s disease pathogenesis: pathways, mechanisms and translational opportunities. Ageing Res Rev 71:101425. 10.1016/j.arr.2021.101425 34384901 10.1016/j.arr.2021.101425

[8] Altaf S, Cummins MJ, Ittner LM, Mattick JS (2025) The emerging roles of long non-coding RNAs in the nervous system. Nat Rev Neurosci 26:661–676. 10.1038/s41583-025-00960-z 40913069 10.1038/s41583-025-00960-z

[9] Ferrer J, Dimitrova N (2024) Transcription regulation by long non-coding RNAs: mechanisms and disease relevance. Nat Rev Mol Cell Biol 25:396–415. 10.1038/s41580-023-00694-9 38242953 10.1038/s41580-023-00694-9 PMC11045326

[10] Gil N, Ulitsky I (2020) Regulation of gene expression by cis-acting long non-coding RNAs. Nat Rev Genet 21:102–117. 10.1038/s41576-019-0184-5 31729473 10.1038/s41576-019-0184-5

[11] Zhang W, Zhao H, Wu Q, Xu W, Xia M (2018) Knockdown of BACE1-AS by siRNA improves memory and learning behaviors in Alzheimer’s disease animal model. Exp Ther Med 16:2080–2086. 10.3892/etm.2018.6359 30186443 10.3892/etm.2018.6359 PMC6122303

[12] Sadick JS, O’Dea MR, Hasel P, Dykstra T, Faustin A, Liddelow SA (2022) Astrocytes and oligodendrocytes undergo subtype-specific transcriptional changes in Alzheimer’s disease. Neuron 110:1788-1805.e10. 10.1016/j.neuron.2022.03.008 35381189 10.1016/j.neuron.2022.03.008 PMC9167747

[13] Fujita M, Gao Z, Zeng L, McCabe C, White CC, Ng B et al (2024) Cell subtype-specific effects of genetic variation in the Alzheimer’s disease brain. Nat Genet 56:605–614. 10.1038/s41588-024-01685-y 38514782 10.1038/s41588-024-01685-y PMC12288883

[14] Leng K, Li E, Eser R, Piergies A, Sit R, Tan M et al (2021) Molecular characterization of selectively vulnerable neurons in Alzheimer’s disease. Nat Neurosci 24:276–287. 10.1038/s41593-020-00764-7 33432193 10.1038/s41593-020-00764-7 PMC7854528

[15] Lau S-F, Cao H, Fu AKY, Ip NY (2020) Single-nucleus transcriptome analysis reveals dysregulation of angiogenic endothelial cells and neuroprotective glia in Alzheimer’s disease. Proc Natl Acad Sci U S A 117:25800–25809. 10.1073/pnas.2008762117 32989152 10.1073/pnas.2008762117 PMC7568283

[16] Giorgi FM, Ceraolo C, Mercatelli D (2022) The R language: an engine for bioinformatics and data science. Life (Basel) 12:648. 10.3390/life12050648 35629316 10.3390/life12050648 PMC9148156

[17] Hao Y, Stuart T, Kowalski MH, Choudhary S, Hoffman P, Hartman A et al (2024) Dictionary learning for integrative, multimodal and scalable single-cell analysis. Nat Biotechnol 42:293–304. 10.1038/s41587-023-01767-y 37231261 10.1038/s41587-023-01767-y PMC10928517

[18] Ianevski A, Giri AK, Aittokallio T (2022) Fully-automated and ultra-fast cell-type identification using specific marker combinations from single-cell transcriptomic data. Nat Commun 13:1246. 10.1038/s41467-022-28803-w 35273156 10.1038/s41467-022-28803-w PMC8913782

[19] Love MI, Huber W, Anders S (2014) Moderated estimation of fold change and dispersion for RNA-seq data with DESeq2. Genome Biol 15:550. 10.1186/s13059-014-0550-8 25516281 10.1186/s13059-014-0550-8 PMC4302049

[20] Leek JT, Johnson WE, Parker HS, Jaffe AE, Storey JD (2012) The sva package for removing batch effects and other unwanted variation in high-throughput experiments. Bioinformatics 28:882–883. 10.1093/bioinformatics/bts034 22257669 10.1093/bioinformatics/bts034 PMC3307112

[21] Wolf FA, Angerer P, Theis FJ (2018) SCANPY: large-scale single-cell gene expression data analysis. Genome Biol 19:15. 10.1186/s13059-017-1382-0 29409532 10.1186/s13059-017-1382-0 PMC5802054

[22] Wolf FA, Hamey FK, Plass M, Solana J, Dahlin JS, Göttgens B et al (2019) Paga: graph abstraction reconciles clustering with trajectory inference through a topology preserving map of single cells. Genome Biol 20:59. 10.1186/s13059-019-1663-x 30890159 10.1186/s13059-019-1663-x PMC6425583

[23] Finak G, McDavid A, Yajima M, Deng J, Gersuk V, Shalek AK et al (2015) MAST: a flexible statistical framework for assessing transcriptional changes and characterizing heterogeneity in single-cell RNA sequencing data. Genome Biol 16:278. 10.1186/s13059-015-0844-5 26653891 10.1186/s13059-015-0844-5 PMC4676162

[24] Zimmerman KD, Espeland MA, Langefeld CD (2021) A practical solution to pseudoreplication bias in single-cell studies. Nat Commun 12:738. 10.1038/s41467-021-21038-1 33531494 10.1038/s41467-021-21038-1 PMC7854630

[25] Haynes W (2013) Benjamini–Hochberg method. Encyclopedia of Systems Biology. New York, NY: Springer New York. pp. 78–78. 10.1007/978-1-4419-9863-7_1215

[26] Mamede I, Queiroz LR, Mata-Machado C, Rodrigues JT, Luscher-Dias T, de Toledo NE et al (2025) Isoformic: a workflow for transcript-level RNA-seq interpretation. NAR Genom Bioinform. 10.1093/nargab/lqaf176 41347231 10.1093/nargab/lqaf176 PMC12673842

[27] Durinck S, Moreau Y, Kasprzyk A, Davis S, De Moor B, Brazma A et al (2005) BioMart and bioconductor: a powerful link between biological databases and microarray data analysis. Bioinformatics 21:3439–3440. 10.1093/bioinformatics/bti525 16082012 10.1093/bioinformatics/bti525

[28] Morabito S, Miyoshi E, Michael N, Shahin S, Martini AC, Head E et al (2021) Single-nucleus chromatin accessibility and transcriptomic characterization of Alzheimer’s disease. Nat Genet 53:1143–1155. 10.1038/s41588-021-00894-z 34239132 10.1038/s41588-021-00894-z PMC8766217

[29] Mathys H, Davila-Velderrain J, Peng Z, Gao F, Mohammadi S, Young JZ et al (2019) Single-cell transcriptomic analysis of Alzheimer’s disease. Nature 570:332–337. 10.1038/s41586-019-1195-2 31042697 10.1038/s41586-019-1195-2 PMC6865822

[30] Anderson AG, Rogers BB, Loupe JM, Rodriguez-Nunez I, Roberts SC, White LM et al (2023) Single nucleus multiomics identifies ZEB1 and MAFB as candidate regulators of Alzheimer’s disease-specific cis-regulatory elements. Cell Genom 3:100263. 10.1016/j.xgen.2023.100263 36950385 10.1016/j.xgen.2023.100263 PMC10025452

[31] Bartas K, Nguyen M, Zhao W, Hui M, Nie Q, Beier KT (2025) Analysis of changes in intercellular communications in Alzheimer’s disease reveals conserved changes in glutamatergic transmission in mice and humans. Sci Rep 15:26248. 10.1038/s41598-025-10795-4 40683902 10.1038/s41598-025-10795-4 PMC12276270

[32] Wang Q, Antone J, Alsop E, Reiman R, Funk C, Bendl J et al (2024) Single cell transcriptomes and multiscale networks from persons with and without Alzheimer’s disease. Nat Commun 15:5815. 10.1038/s41467-024-49790-0 38987616 10.1038/s41467-024-49790-0 PMC11237088

[33] Carlson M, Pagès H, Aboyoun P, Falcon S, Morgan M, Sarkar D, Lawrence M (2017) GenomicFeatures. Bioconductor. 10.18129/B9.BIOC.GENOMICFEATURES

[34] Harrell FE Jr (2003) Hmisc: Harrell Miscellaneous. CRAN: Contributed Packages. The R Foundation. 10.32614/cran.package.hmisc

[35] Casper J, Speir ML, Raney BJ, Perez G, Nassar LR, Lee CM et al (2026) The UCSC Genome browser database: 2026 update. Nucleic Acids Res 54:D1331–D1335. 10.1093/nar/gkaf1250 41251146 10.1093/nar/gkaf1250 PMC12807699

[36] Fixemer S, Ameli C, Hammer G, Salamanca L, Uriarte Huarte O, Schwartz C et al (2022) Microglia phenotypes are associated with subregional patterns of concomitant tau, amyloid-β and α-synuclein pathologies in the hippocampus of patients with Alzheimer’s disease and dementia with Lewy bodies. Acta Neuropathol Commun 10:36. 10.1186/s40478-022-01342-7 35296366 10.1186/s40478-022-01342-7 PMC8925098

[37] Bryant A, Li Z, Jayakumar R, Serrano-Pozo A, Woost B, Hu M et al (2023) Endothelial cells are heterogeneous in different brain regions and are dramatically altered in Alzheimer’s disease. J Neurosci 43:4541–4557. 10.1523/JNEUROSCI.0237-23.2023 37208174 10.1523/JNEUROSCI.0237-23.2023 PMC10278684

[38] Kaufman SK, Sanders DW, Thomas TL, Ruchinskas AJ, Vaquer-Alicea J, Sharma AM et al (2016) Tau prion strains dictate patterns of cell pathology, progression rate, and regional vulnerability in vivo. Neuron 92:796–812. 10.1016/j.neuron.2016.09.055 27974162 10.1016/j.neuron.2016.09.055 PMC5392364

[39] Byun MS, Kim SE, Park J, Yi D, Choe YM, Sohn BK et al (2015) Heterogeneity of regional brain atrophy patterns associated with distinct progression rates in Alzheimer’s disease. PLoS ONE 10:e0142756. 10.1371/journal.pone.0142756 26618360 10.1371/journal.pone.0142756 PMC4664412

[40] Birkenbihl C, Salimi Y, Fröhlich H (2022) Japanese Alzheimer’s Disease Neuroimaging Initiative, Alzheimer’s Disease Neuroimaging Initiative. Unraveling the heterogeneity in Alzheimer’s disease progression across multiple cohorts and the implications for data-driven disease modeling. Alzheimers Dement 18: 251–261. 10.1002/alz.12387 10.1002/alz.12387 PMC13262070 34109729

[41] Sirkis DW, Bonham LW, Johnson TP, La Joie R, Yokoyama JS (2022) Dissecting the clinical heterogeneity of early-onset Alzheimer’s disease. Mol Psychiatry 27:2674–2688. 10.1038/s41380-022-01531-9 35393555 10.1038/s41380-022-01531-9 PMC9156414

[42] Liu SJ, Nowakowski TJ, Pollen AA, Lui JH, Horlbeck MA, Attenello FJ et al (2016) Single-cell analysis of long non-coding RNAs in the developing human neocortex. Genome Biol 17:67. 10.1186/s13059-016-0932-1 27081004 10.1186/s13059-016-0932-1 PMC4831157

[43] He Z, Lan Y, Zhou X, Yu B, Zhu T, Yang F et al (2024) Single-cell transcriptome analysis dissects lncRNA-associated gene networks in Arabidopsis. Plant Commun 5:100717. 10.1016/j.xplc.2023.100717 37715446 10.1016/j.xplc.2023.100717 PMC10873878

[44] Mattick JS, Amaral PP, Carninci P, Carpenter S, Chang HY, Chen L-L et al (2023) Long non-coding RNAs: definitions, functions, challenges and recommendations. Nat Rev Mol Cell Biol 24:430–447. 10.1038/s41580-022-00566-8 36596869 10.1038/s41580-022-00566-8 PMC10213152

[45] Grammatikakis I, Lal A (2022) Significance of lncrna abundance to function. Mamm Genome 33:271–280. 10.1007/s00335-021-09901-4 34406447 10.1007/s00335-021-09901-4

[46] Pasquini G, Rojo Arias JE, Schäfer P, Busskamp V (2021) Automated methods for cell type annotation on scrna-seq data. Comput Struct Biotechnol J 19:961–969. 10.1016/j.csbj.2021.01.015 33613863 10.1016/j.csbj.2021.01.015 PMC7873570

[47] Darmanis S, Sloan SA, Zhang Y, Enge M, Caneda C, Shuer LM et al (2015) A survey of human brain transcriptome diversity at the single cell level. Proc Natl Acad Sci U S A 112:7285–7290. 10.1073/pnas.1507125112 26060301 10.1073/pnas.1507125112 PMC4466750

[48] Zhang Y, Sloan SA, Clarke LE, Caneda C, Plaza CA, Blumenthal PD et al (2016) Purification and characterization of progenitor and mature human astrocytes reveals transcriptional and functional differences with mouse. Neuron 89:37–53. 10.1016/j.neuron.2015.11.013 26687838 10.1016/j.neuron.2015.11.013 PMC4707064

[49] Zhang Y, Chen K, Sloan SA, Bennett ML, Scholze AR, O’Keeffe S et al (2014) An RNA-sequencing transcriptome and splicing database of glia, neurons, and vascular cells of the cerebral cortex. J Neurosci 34:11929–11947. 10.1523/JNEUROSCI.1860-14.2014 25186741 10.1523/JNEUROSCI.1860-14.2014 PMC4152602

[50] Baazaoui N, Y Alfaifi M, Ben Saad R, Garzoli S (2025) Potential role of long noncoding RNA maternally expressed gene 3 (MEG3) in the process of neurodegeneration. Neuroscience 565:487–498. 10.1016/j.neuroscience.2024.12.023 39675694 10.1016/j.neuroscience.2024.12.023

[51] Mus E, Hof PR, Tiedge H (2007) Dendritic BC200 RNA in aging and in Alzheimer’s disease. Proc Natl Acad Sci U S A 104:10679–10684. 10.1073/pnas.0701532104 17553964 10.1073/pnas.0701532104 PMC1965572

[52] Boche D, Gordon MN (2022) Diversity of transcriptomic microglial phenotypes in aging and Alzheimer’s disease. Alzheimers Dement 18:360–376. 10.1002/alz.12389 34223696 10.1002/alz.12389 PMC9059230

[53] Yu Y, Mai Y, Zheng Y, Shi L (2024) Assessing and mitigating batch effects in large-scale omics studies. Genome Biol 25:254. 10.1186/s13059-024-03401-9 39363244 10.1186/s13059-024-03401-9 PMC11447944

[54] Barrett A, Varol E, Weinreb A, Taylor SR, McWhirter RM, Cros C et al (2025) Integrating bulk and single cell RNA-seq refines transcriptomic profiles of individual C. elegans neurons. 10.7554/elife.106183.1

[55] Yu X, Abbas-Aghababazadeh F, Chen YA, Fridley BL (2021) Statistical and bioinformatics analysis of data from bulk and single-cell RNA sequencing experiments. Methods Mol Biol 2194:143–175. 10.1007/978-1-0716-0849-4_9 32926366 10.1007/978-1-0716-0849-4_9 PMC7771369

[56] Deveson IW, Hardwick SA, Mercer TR, Mattick JS (2017) The dimensions, dynamics, and relevance of the mammalian noncoding transcriptome. Trends Genet 33:464–478. 10.1016/j.tig.2017.04.004 28535931 10.1016/j.tig.2017.04.004

[57] Uszczynska-Ratajczak B, Lagarde J, Frankish A, Guigó R, Johnson R (2018) Towards a complete map of the human long non-coding RNA transcriptome. Nat Rev Genet 19:535–548. 10.1038/s41576-018-0017-y 29795125 10.1038/s41576-018-0017-y PMC6451964

[58] Hardwick SA, Chen WY, Wong T, Deveson IW, Blackburn J, Andersen SB et al (2016) Spliced synthetic genes as internal controls in RNA sequencing experiments. Nat Methods 13:792–798. 10.1038/nmeth.3958 27502218 10.1038/nmeth.3958

[59] Grubman A, Chew G, Ouyang JF, Sun G, Choo XY, McLean C et al (2019) A single-cell atlas of entorhinal cortex from individuals with Alzheimer’s disease reveals cell-type-specific gene expression regulation. Nat Neurosci 22:2087–2097. 10.1038/s41593-019-0539-4 31768052 10.1038/s41593-019-0539-4

[60] Ohashi K (2015) Roles of cofilin in development and its mechanisms of regulation. Dev Growth Differ 57:275–290. 10.1111/dgd.12213 25864508 10.1111/dgd.12213

[61] Bahader GA, James AW, Almarghalani DA, Shah ZA (2023) Cofilin inhibitor protects against traumatic brain injury-induced oxidative stress and neuroinflammation. Biology (Basel) 12:630. 10.3390/biology12040630 37106830 10.3390/biology12040630 PMC10136258

[62] Alhadidi Q, Shah ZA (2018) Cofilin mediates LPS-induced microglial cell activation and associated neurotoxicity through activation of NF-κB and JAK-STAT pathway. Mol Neurobiol 55:1676–1691. 10.1007/s12035-017-0432-7 28194647 10.1007/s12035-017-0432-7 PMC5554748

[63] Shah N, Kasture AS, Fischer FP, Sitte HH, Hummel T, Sucic S (2024) A transporter’s doom or destiny: SLC6A1 in health and disease, novel molecular targets and emerging therapeutic prospects. Front Mol Neurosci 17:1466694. 10.3389/fnmol.2024.1466694 39268250 10.3389/fnmol.2024.1466694 PMC11390516

[64] Carvill GL, McMahon JM, Schneider A, Zemel M, Myers CT, Saykally J et al (2015) Mutations in the GABA transporter SLC6A1 cause epilepsy with myoclonic-atonic seizures. Am J Hum Genet 96:808–815. 10.1016/j.ajhg.2015.02.016 25865495 10.1016/j.ajhg.2015.02.016 PMC4570550

[65] Ung DC, Martin S, Hérault Y, Laumonnier F (2025) The PTCHD1 protein: a prominent actor in brain function and in neurodevelopmental disorders. Neurosci Biobehav Rev 176:106307. 10.1016/j.neubiorev.2025.106307 40730316 10.1016/j.neubiorev.2025.106307

[66] Ung DC, Iacono G, Méziane H, Blanchard E, Papon M-A, Selten M et al (2018) Ptchd1 deficiency induces excitatory synaptic and cognitive dysfunctions in mouse. Mol Psychiatry 23:1356–1367. 10.1038/mp.2017.39 28416808 10.1038/mp.2017.39 PMC5984103

[67] Xu D, Zhang C, Bi X, Xu J, Guo S, Li P et al (2024) Mapping enhancer and chromatin accessibility landscapes charts the regulatory network of Alzheimer’s disease. Comput Biol Med 168:107802. 10.1016/j.compbiomed.2023.107802 38056211 10.1016/j.compbiomed.2023.107802

[68] Zhong X, Cordeddu L, Gamboa-Cedeno A, Bengtzén S, Ekwall K, Lennartsson A et al (2025) Disease-specific epigenetic deregulation of enhancers, transposons, and polycomb targets in acute promyelocytic leukemia. Genome Med 17:135. 10.1186/s13073-025-01565-y 41168841 10.1186/s13073-025-01565-y PMC12573822

[69] Morlando M, Fatica A (2018) Alteration of epigenetic regulation by long noncoding RNAs in cancer. Int J Mol Sci 19:570. 10.3390/ijms19020570 29443889 10.3390/ijms19020570 PMC5855792

[70] Dwivedi Y, Roy B (2025) Aberrant expression of long non-coding RNAs and their regulatory role in chromatin-mediated gene expression changes in the prefrontal cortex of major depressive disorder subjects. Mol Psychiatry. 10.1038/s41380-025-03396-0 41413201 10.1038/s41380-025-03396-0 PMC13099372

[71] Katano T, Konno K, Takao K, Abe M, Yoshikawa A, Miyakawa T et al (2023) Brain-enriched guanylate kinase-associated protein, a component of the post-synaptic density protein complexes, contributes to learning and memory. Sci Rep 13:22027. 10.1038/s41598-023-49537-9 38086879 10.1038/s41598-023-49537-9 PMC10716515

[72] Tylek K, Basta-Kaim A (2025) Emerging role of oligodendrocytes malfunction in the progression of Alzheimer’s disease. J Neuroimmune Pharmacol 20:79. 10.1007/s11481-025-10236-z 40887534 10.1007/s11481-025-10236-z PMC12399730

[73] Guo S, Yu X, Zhang H (2025) The role of oligodendrocytes in Alzheimer’s disease pathogenesis and therapy. Neuroglia 6:46. 10.3390/neuroglia6040046

[74] Gomez PT, Carver CM, Rodriguez SL, Wang L, Zhang X, Schafer MJ (2024) Aging and senescent fates of oligodendrocyte precursor cells in the mouse brain. NPJ Aging 10:47. 10.1038/s41514-024-00176-y 39438481 10.1038/s41514-024-00176-y PMC11496697

[75] Dimovasili C, Fair AE, Garza IR, Batterman KV, Mortazavi F, Moore TL et al (2023) Aging compromises oligodendrocyte precursor cell maturation and efficient remyelination in the monkey brain. GeroScience 45:249–264. 10.1007/s11357-022-00621-4 35930094 10.1007/s11357-022-00621-4 PMC9886778

[76] Behrendt G, Baer K, Buffo A, Curtis MA, Faull RL, Rees MI et al (2013) Dynamic changes in myelin aberrations and oligodendrocyte generation in chronic amyloidosis in mice and men. Glia 61:273–286. 10.1002/glia.22432 23090919 10.1002/glia.22432

[77] Ferreira S, Pitman KA, Wang S, Summers BS, Bye N, Young KM et al (2020) Amyloidosis is associated with thicker myelin and increased oligodendrogenesis in the adult mouse brain. J Neurosci Res 98:1905–1932. 10.1002/jnr.24672 32557778 10.1002/jnr.24672 PMC7540704

[78] Huang Z, Zhang Y, Zou P, Zong X, Zhang Q (2025) Myelin dysfunction in aging and brain disorders: mechanisms and therapeutic opportunities. Mol Neurodegener 20:69. 10.1186/s13024-025-00861-w 40518508 10.1186/s13024-025-00861-w PMC12168329

[79] Xie Y, Chen L, Wang L, Liu T, Zheng Y, Si L et al (2024) Single-nucleus transcriptomic analysis reveals the relationship between gene expression in oligodendrocyte lineage and major depressive disorder. J Transl Med 22:109. 10.1186/s12967-023-04727-x 38281050 10.1186/s12967-023-04727-x PMC10822185

[80] West JA, Davis CP, Sunwoo H, Simon MD, Sadreyev RI, Wang PI et al (2014) The long noncoding RNAs NEAT1 and MALAT1 bind active chromatin sites. Mol Cell 55:791–802. 10.1016/j.molcel.2014.07.012 25155612 10.1016/j.molcel.2014.07.012 PMC4428586

[81] Huang M, Wang H, Hu X, Cao X (2019) LncRNA MALAT1 binds chromatin remodeling subunit BRG1 to epigenetically promote inflammation-related hepatocellular carcinoma progression. Oncoimmunology 8:e1518628. 10.1080/2162402X.2018.1518628 30546959 10.1080/2162402X.2018.1518628 PMC6287787

