# Supplementary figures and images for: Uncovering Spatiotemporal and Functional Dynamics of Long Non-coding RNAs During Alzheimer’s Progression in the Human Brain at Single-Cell Resolution
Source: Mol Neurobiol. 2026 Apr 30;63(1):599. doi: 10.1007/s12035-026-05859-z (PMC13132975; doi:10.1007/s12035-026-05859-z)

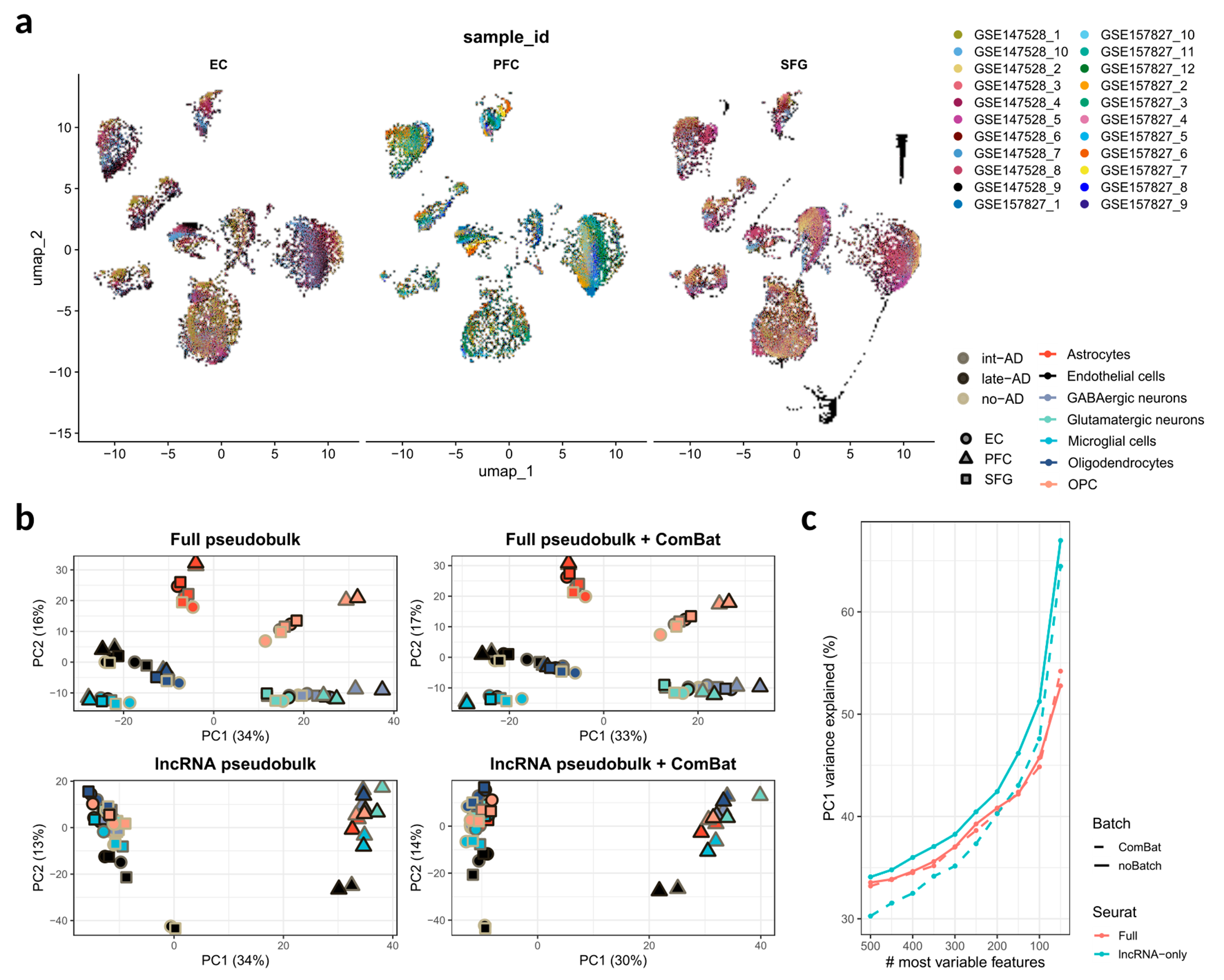

Supplement: Supplementary file 1 — Batch effect correction. (A) Dimensionality-reduced representation of all detected cells from both datasets, visualized by sample ID. (B) Pseudobulk PCA of all cell type-brain region-AD stage combinations using the full transcriptome and lncRNA-only datasets, with and without batch correction. Cell types are indicated by color, brain region by point shape, and AD stage by border color. (C) Variance explained by PC1 across reducing numbers of top variable features. Solid lines represent uncorrected data, dashed lines represent batch-corrected data; colors allow to distinguish between full transcriptome and lncRNA-only datasets (PNG 1.34 MB) [file 12035_2026_5859_Fig7_ESM.png]

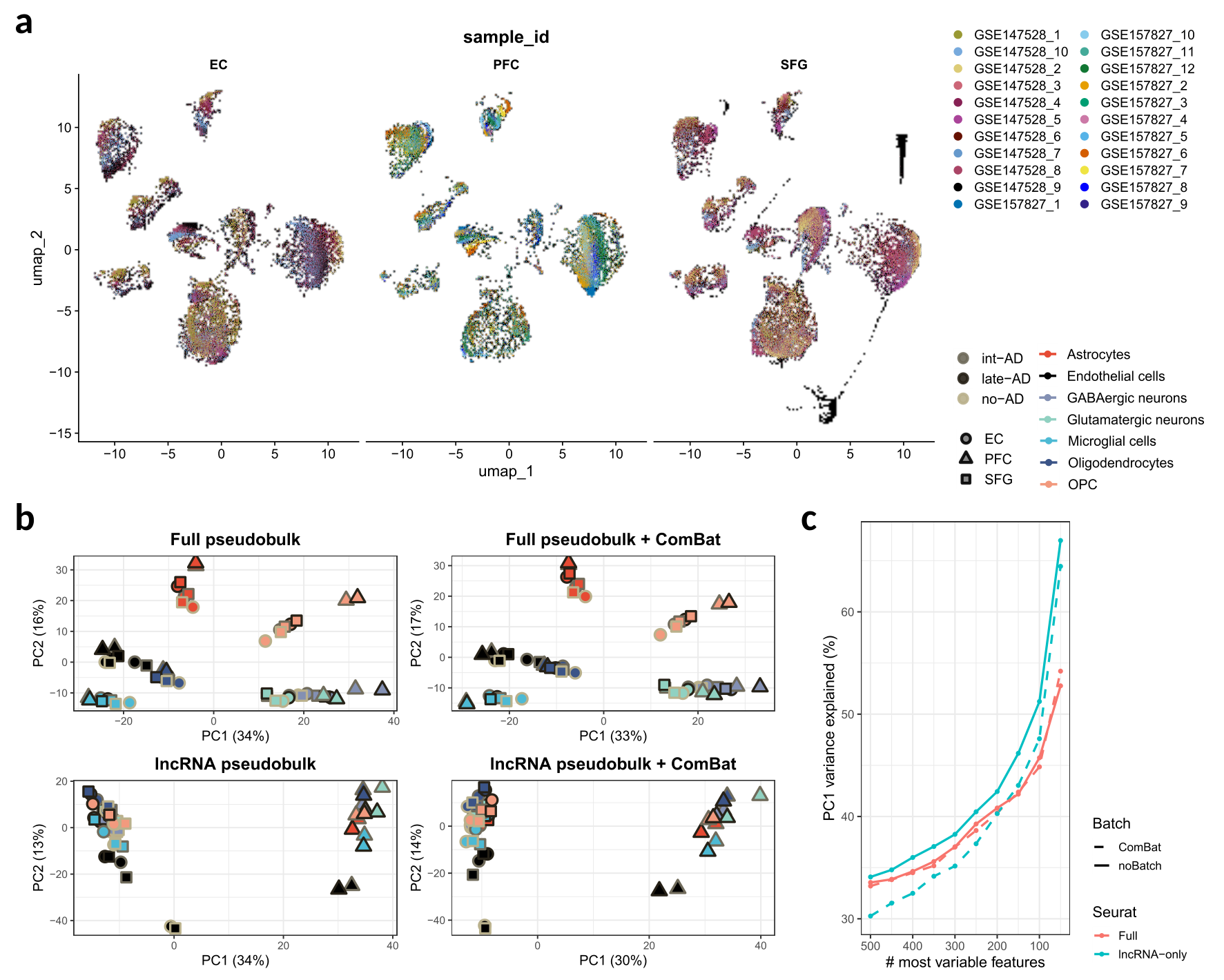

Supplement: Supplementary file 2 — High resolution image (TIFF 778 KB) [file 12035_2026_5859_MOESM1_ESM.tiff]

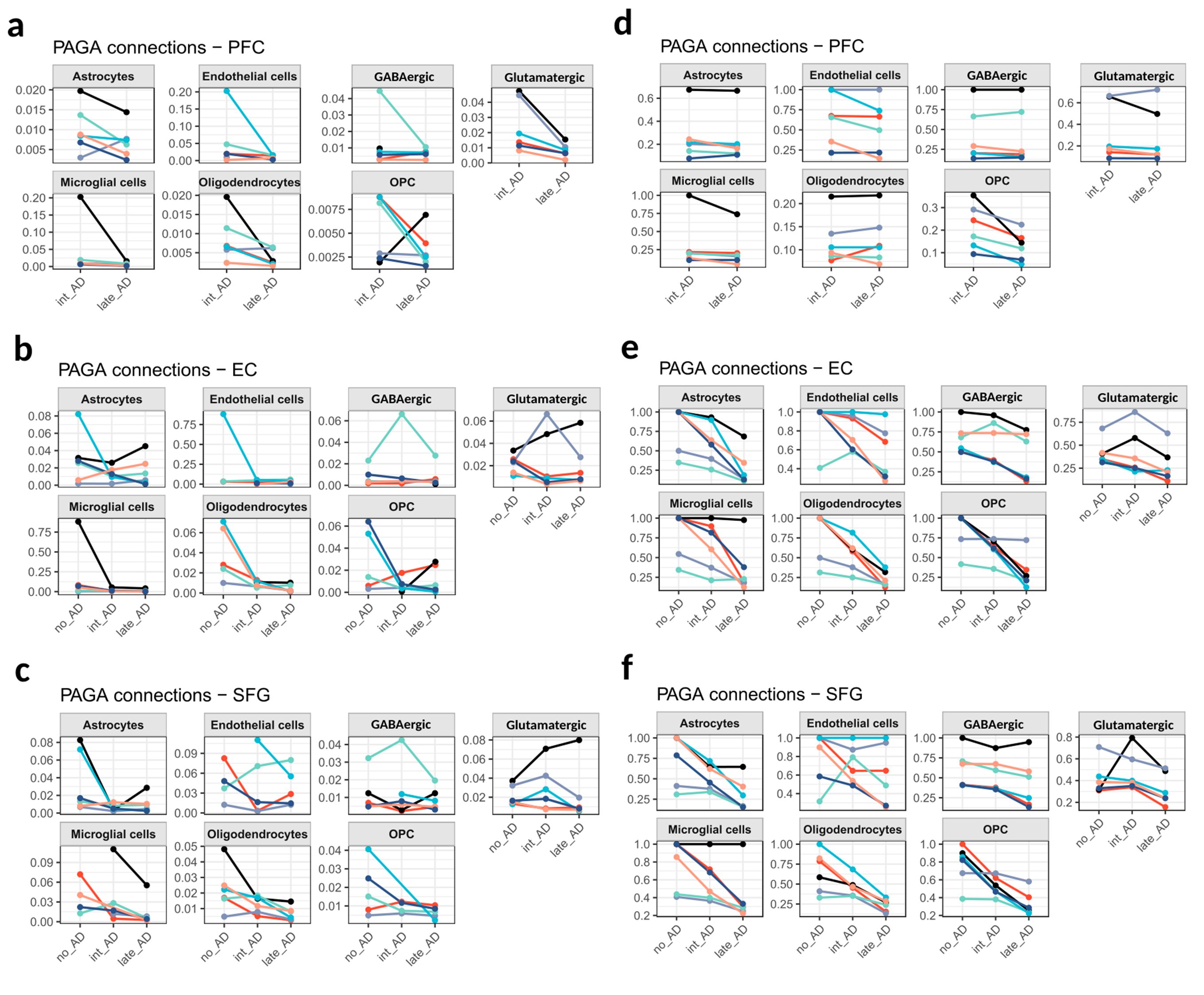

Supplement: Supplementary file 3 — Variation in intercellular transcriptomic similarity. (A-C) Full transcriptome-derived PAGA-extracted intercellular connection variations across AD stages within prefrontal cortex, entorhinal cortex and superior frontal gyrus, respectively. (D-F) lncRNA-only PAGA-extracted intercellular connection variations across AD stages within prefrontal cortex, entorhinal cortex and superior frontal gyrus, respectively (PNG 1.62 MB) [file 12035_2026_5859_Fig8_ESM.png]

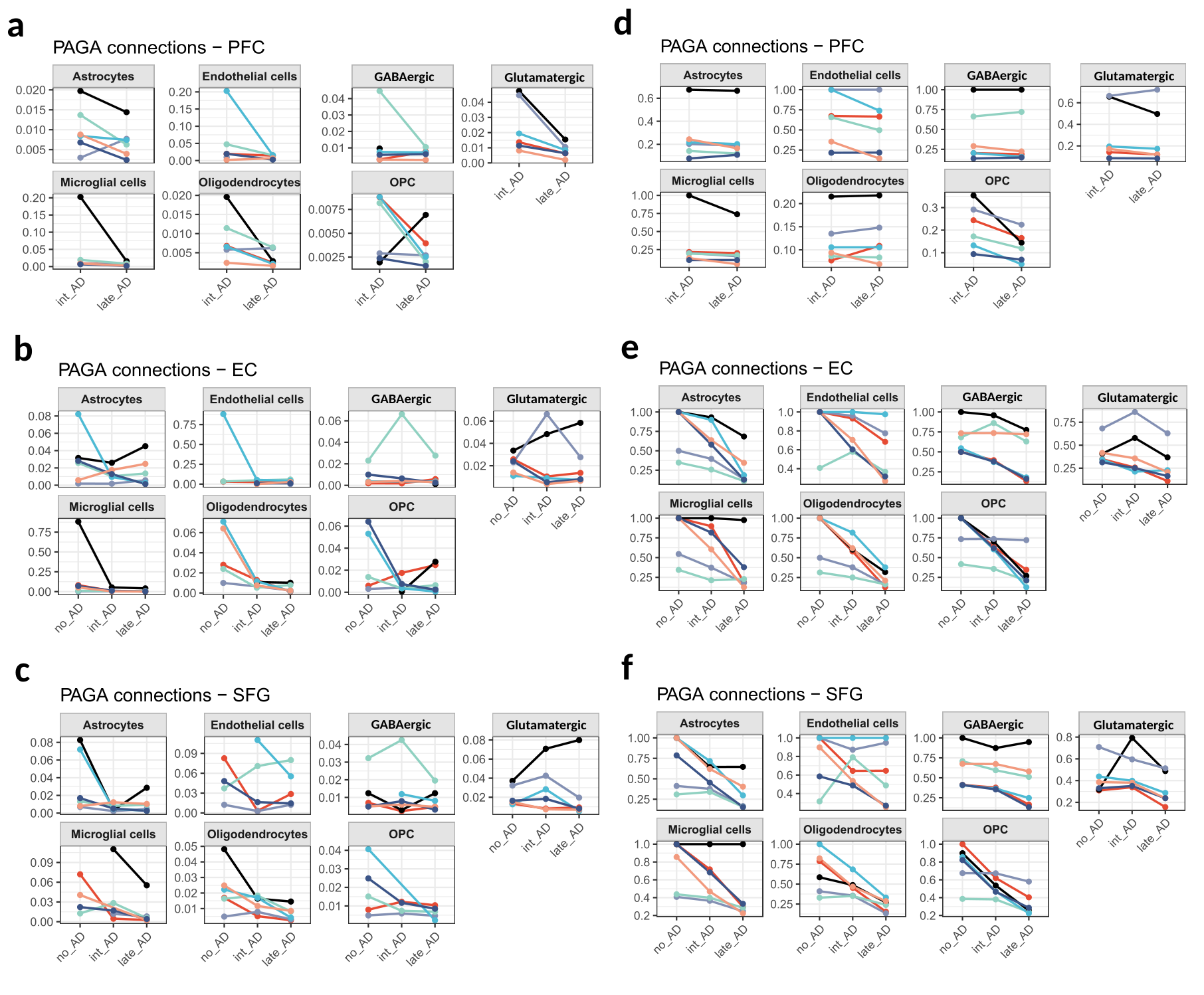

Supplement: Supplementary file 4 — High resolution image (TIFF 693 KB) [file 12035_2026_5859_MOESM2_ESM.tiff]

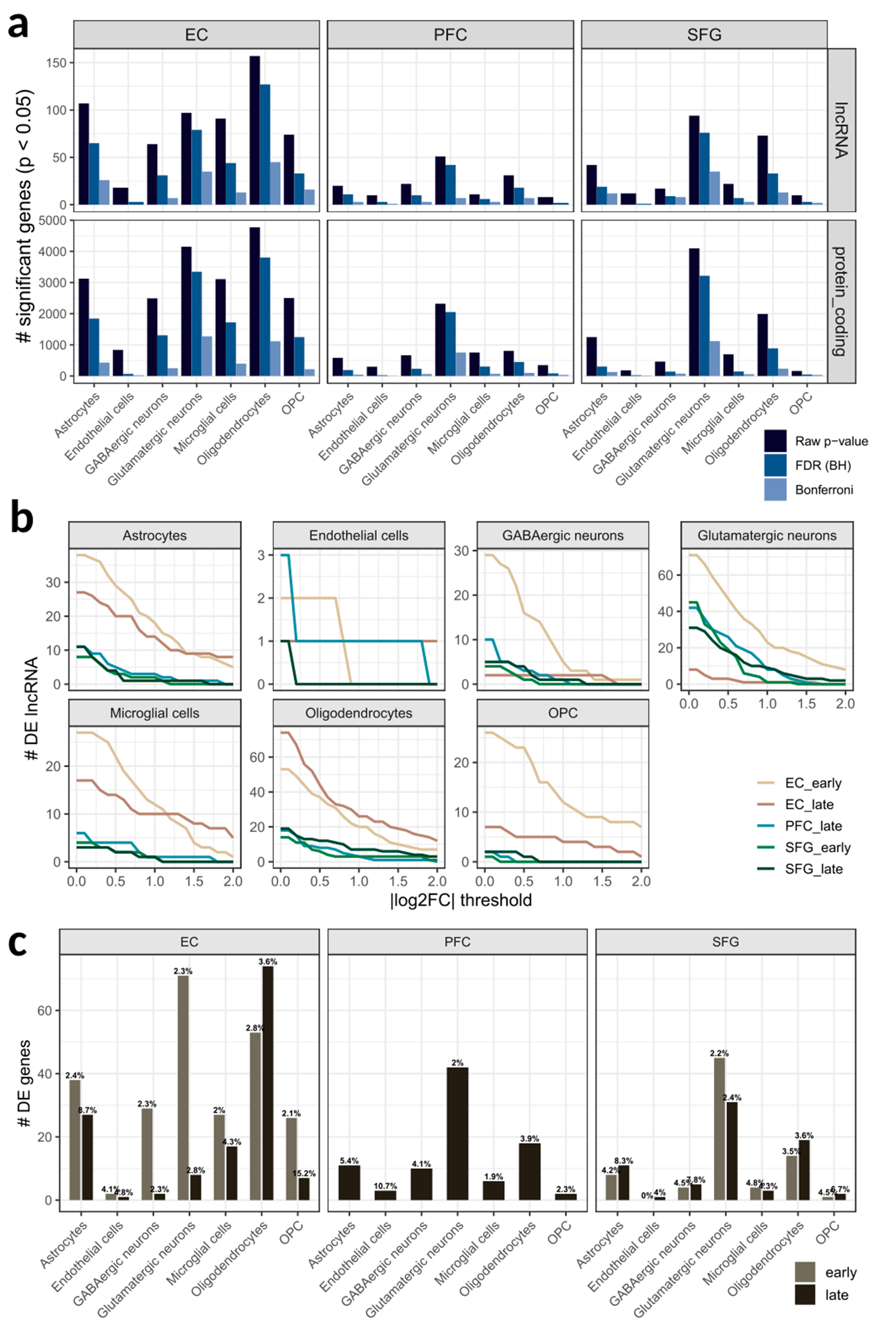

Supplement: Supplementary file 5 — Thresholds for differential expression of lncRNAs. (A) Number of significant DEGs per cell type and region according to different p-value adjustment methods. (B) Number of differentially expressed lncRNAs across log2 fold-change thresholds. (C) Proportion of the altered transcriptome represented by lncRNAs across cell types, faceted by tissue. Bar colors indicate AD stage; the y-axis also reflects absolute counts of dysregulated lncRNAs (PNG 759 KB) [file 12035_2026_5859_Fig9_ESM.png]

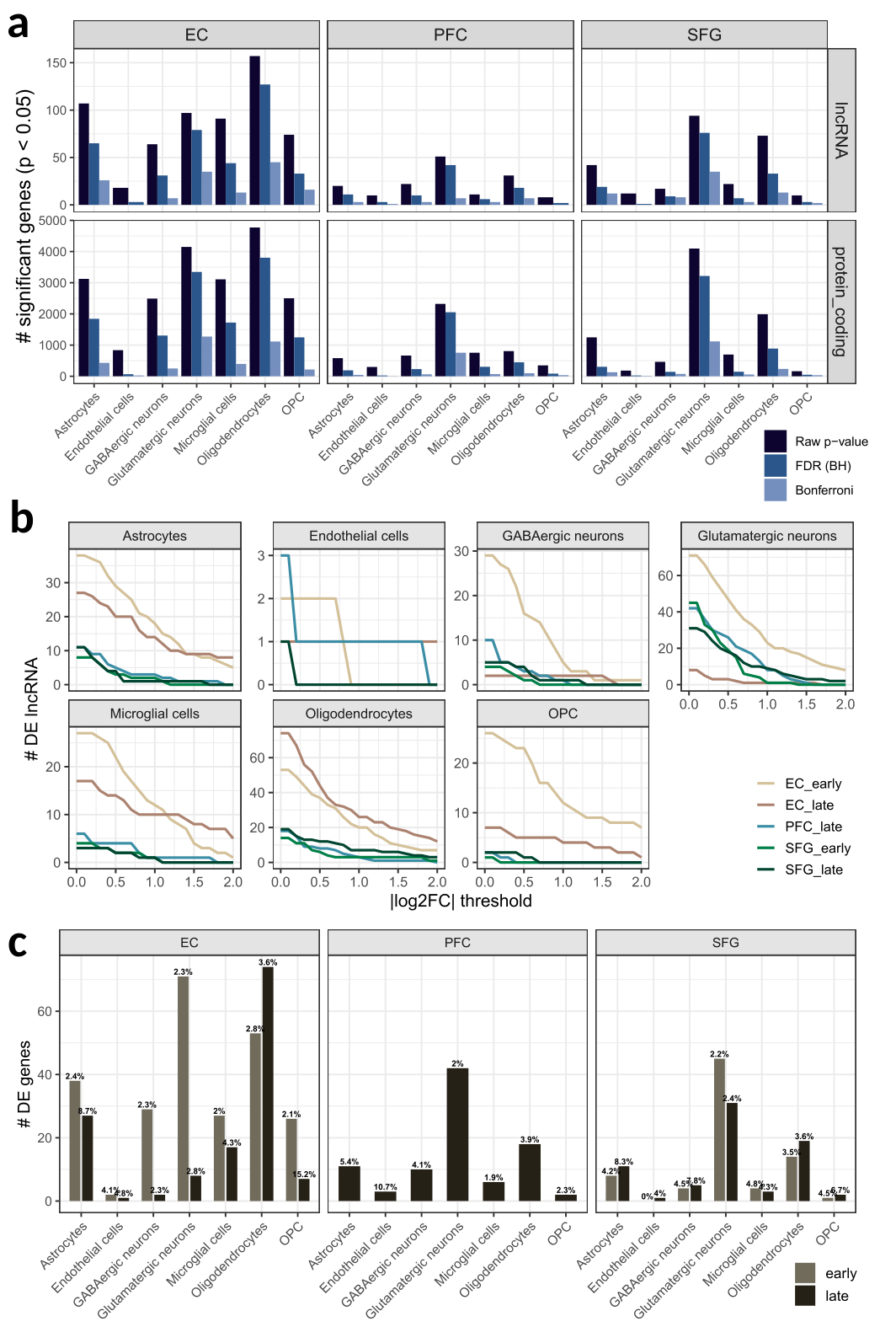

Supplement: Supplementary file 6 — High resolution image (TIFF 416 KB) [file 12035_2026_5859_MOESM3_ESM.tiff]

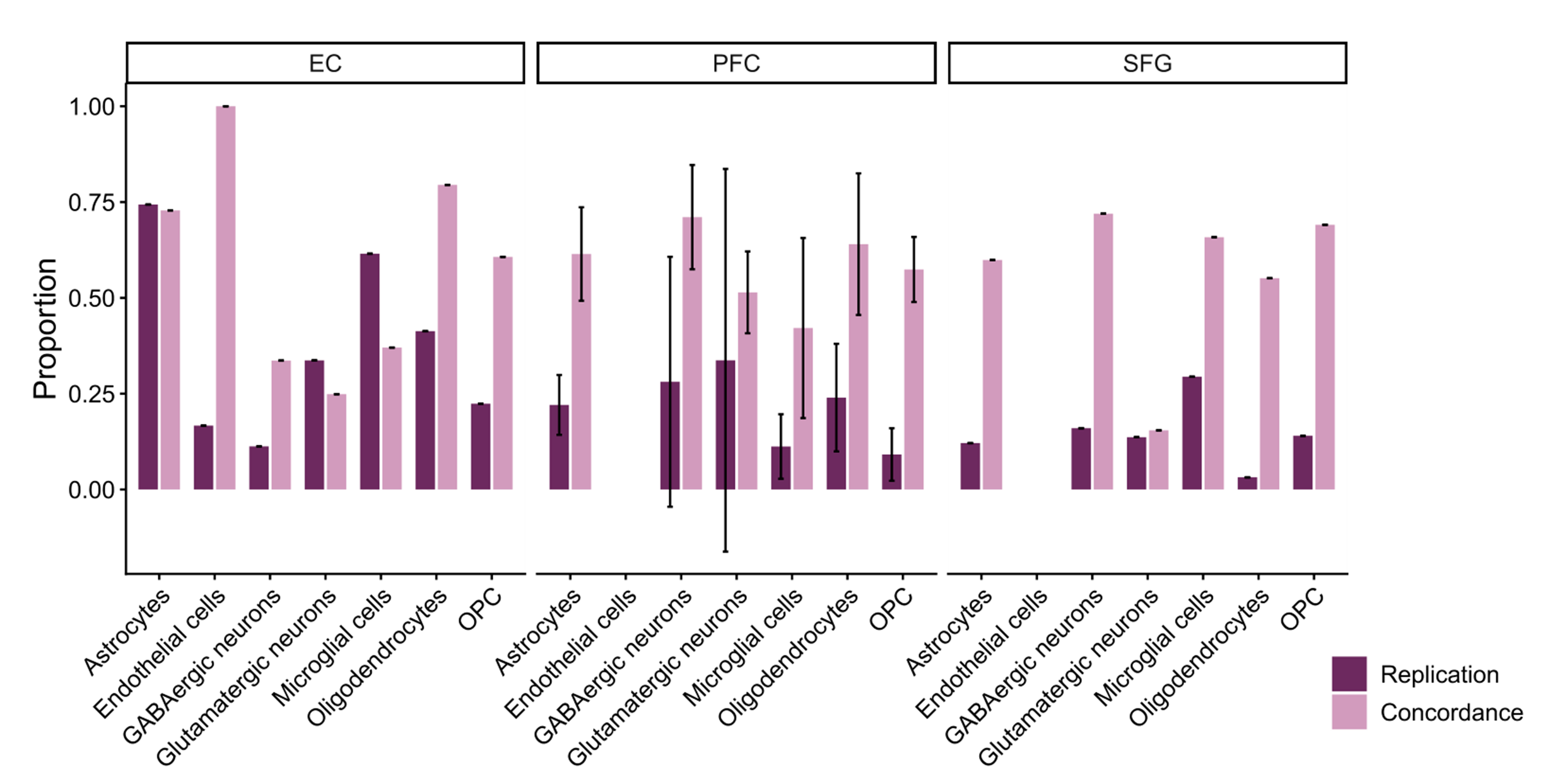

Supplement: Supplementary file 7 — Cross-study comparison of differential expression results. For each cell type within a given tissue, replication was defined as the number of overlapping significant DEGs between pairwise comparisons across studies. Concordance was defined as the proportion of replicated DEGs showing the same direction of expression change (PNG 358 KB) [file 12035_2026_5859_Fig10_ESM.png]

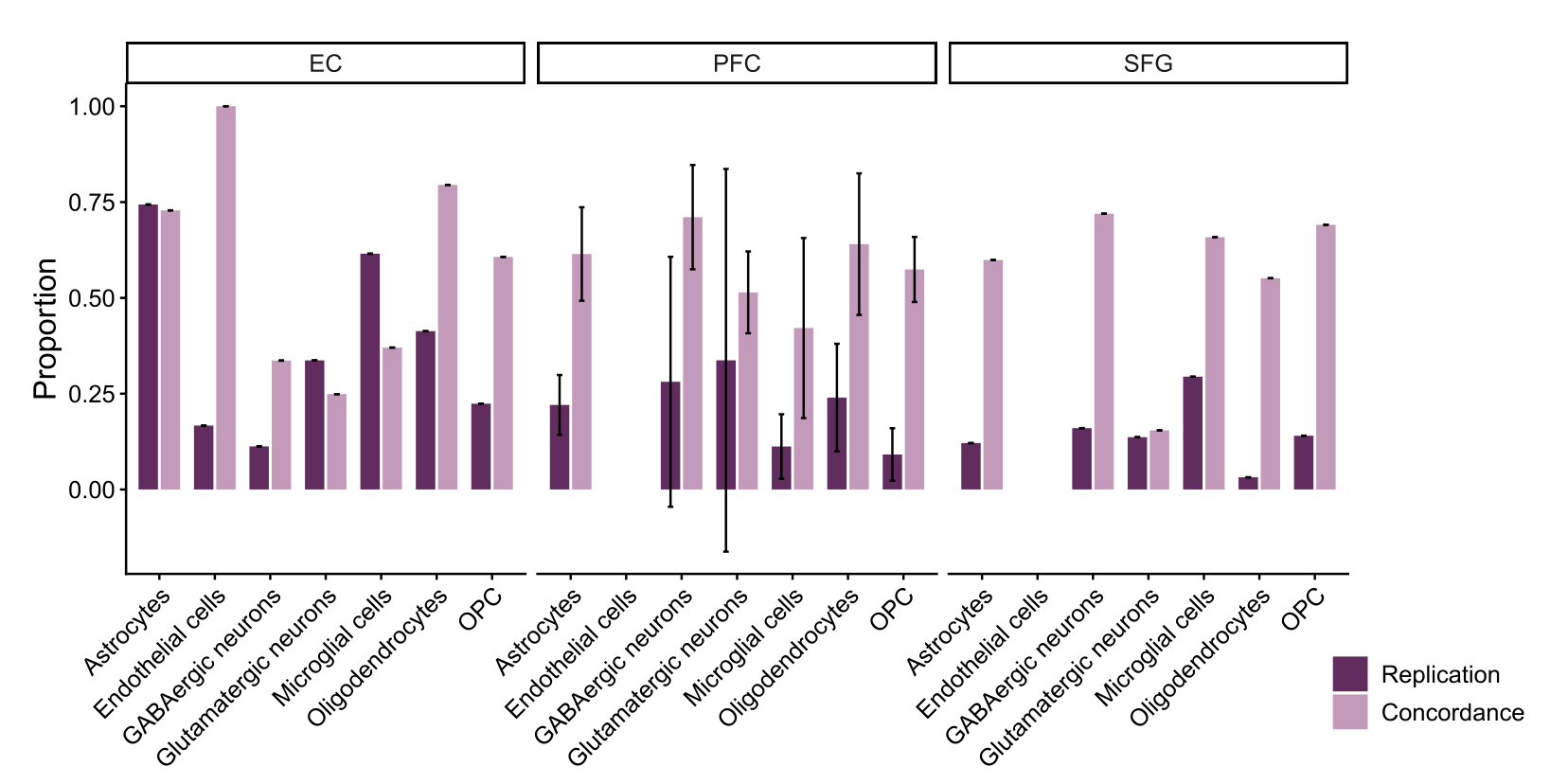

Supplement: Supplementary file 8 — High resolution image (TIFF 170 KB) [file 12035_2026_5859_MOESM4_ESM.tiff]

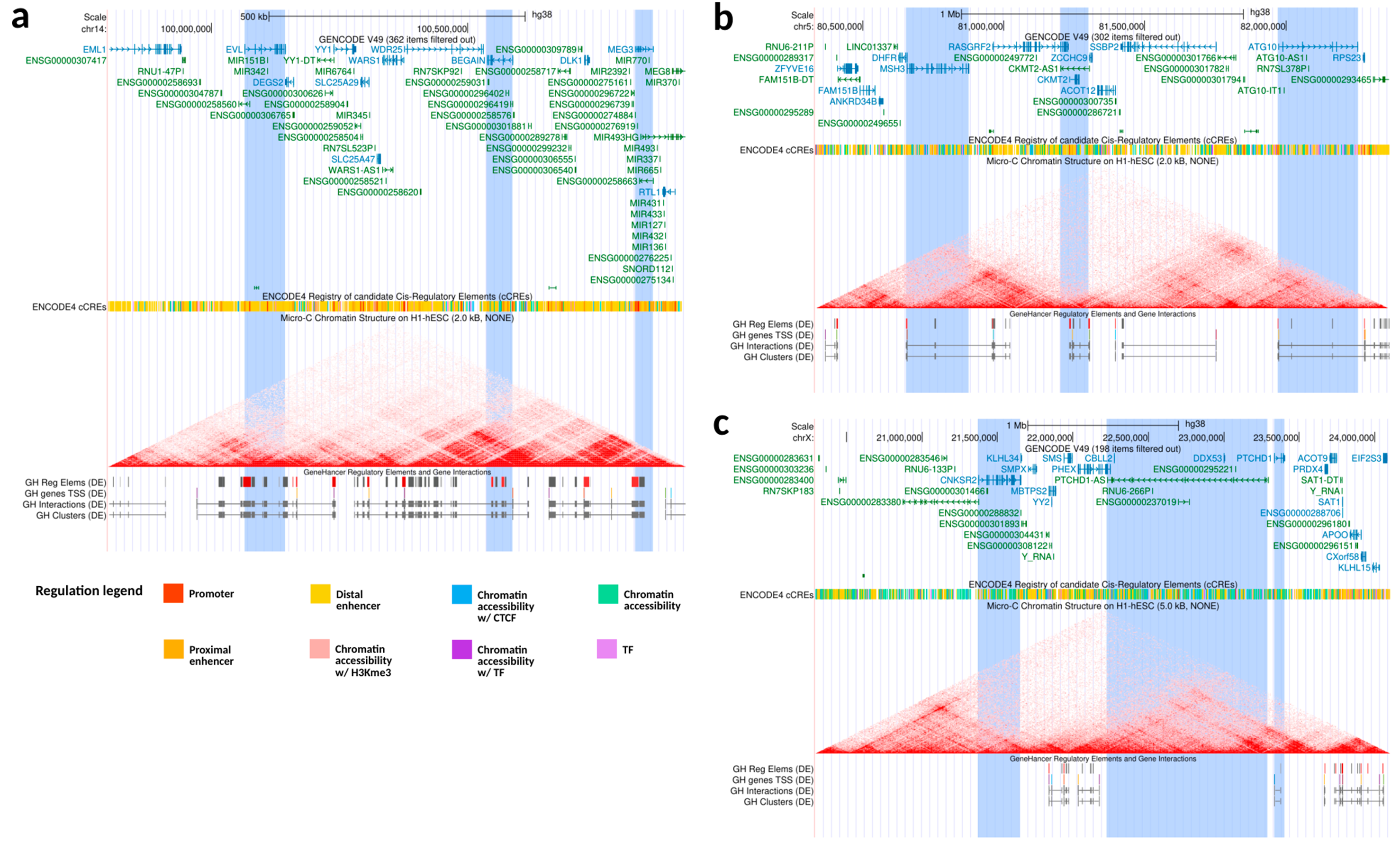

Supplement: Supplementary file 9 — Genomic regions with multiple correlated genes. (A-C) Genomic loci of (A) MEG3, (B) CKMT2-AS1, and (C) PTCHD1-AS. Tracks were obtained from the UCSC Genome Browser (GENCODE v49, ENCODE cCREs, GeneHancer, Hi-C/Micro-C). (http://genome.ucsc.edu). Highlighted genes indicate lncRNAs and protein-coding genes with correlated expression (PNG 2.13 MB) [file 12035_2026_5859_Fig11_ESM.png]

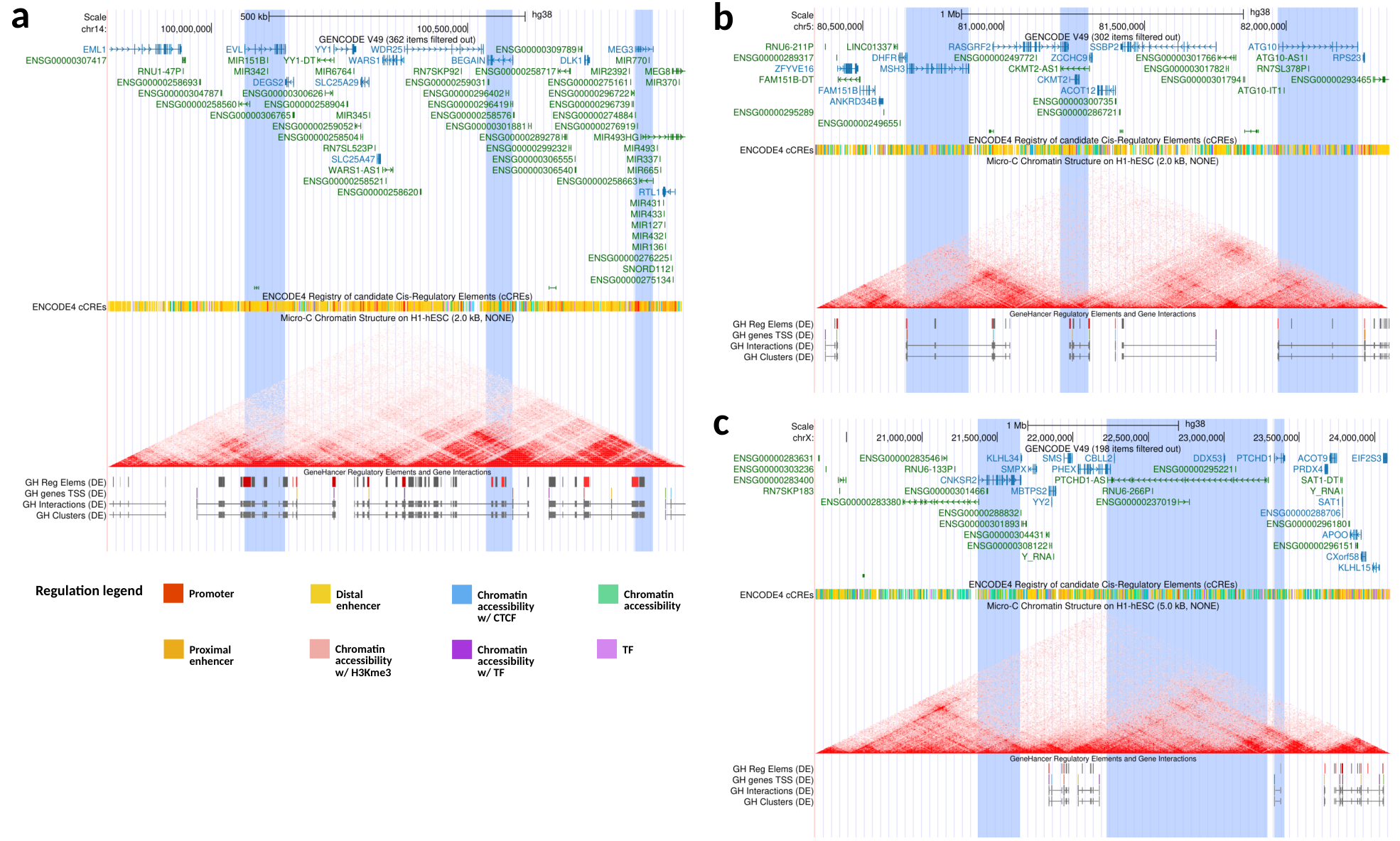

Supplement: Supplementary file 10 — High resolution image (TIFF 1.87 MB) [file 12035_2026_5859_MOESM5_ESM.tiff]

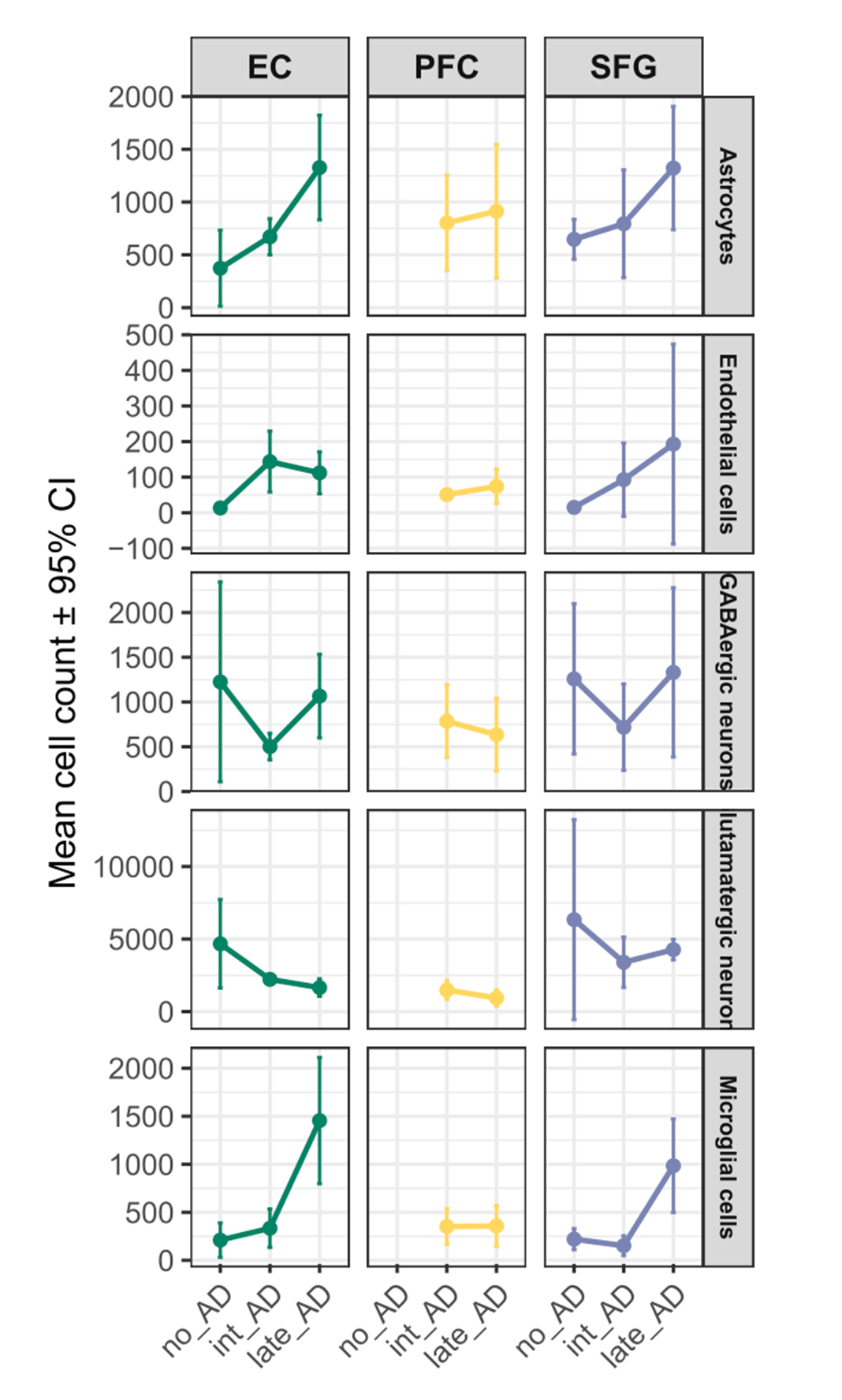

Supplement: Supplementary file 11 — Cell-type abundance across AD progression. Abundance of astrocytes, endothelial cells, GABAergic neurons, glutamatergic neurons and microglia across brain regions and AD stages. Each dot represents the mean number of cells per sample; error bars show 95% confidence intervals (PNG 334 KB) [file 12035_2026_5859_Fig12_ESM.png]

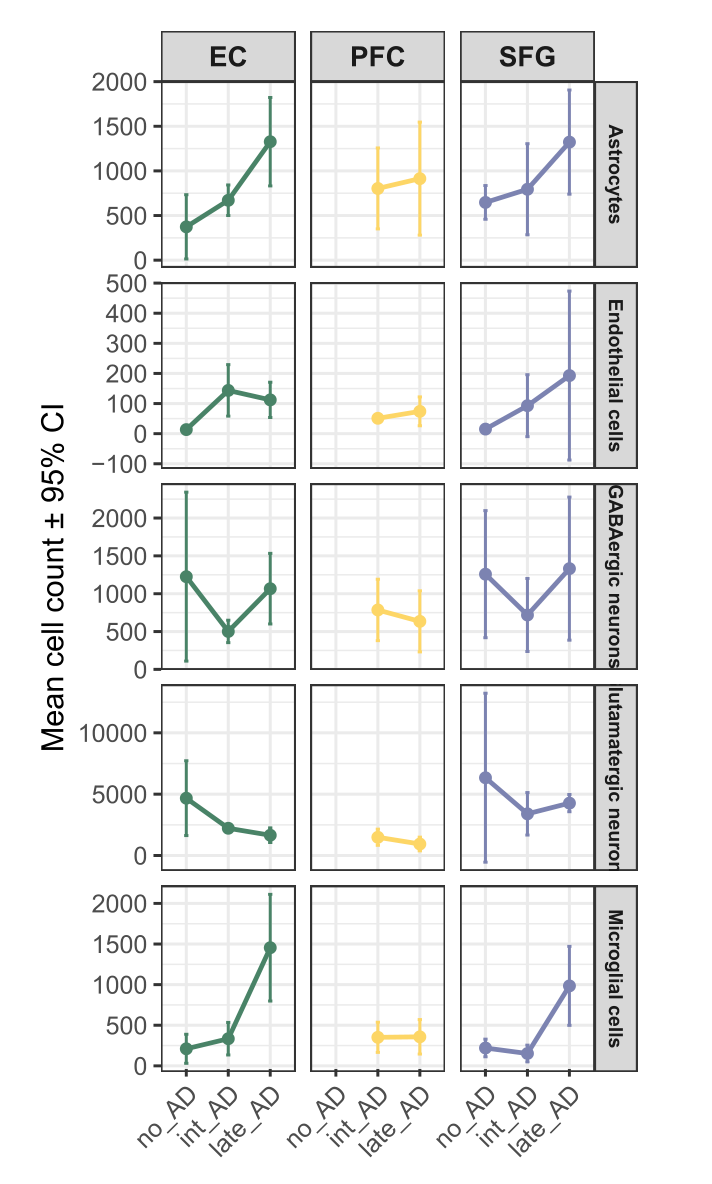

Supplement: Supplementary file 12 — High resolution image (TIFF 181 KB) [file 12035_2026_5859_MOESM6_ESM.tiff]

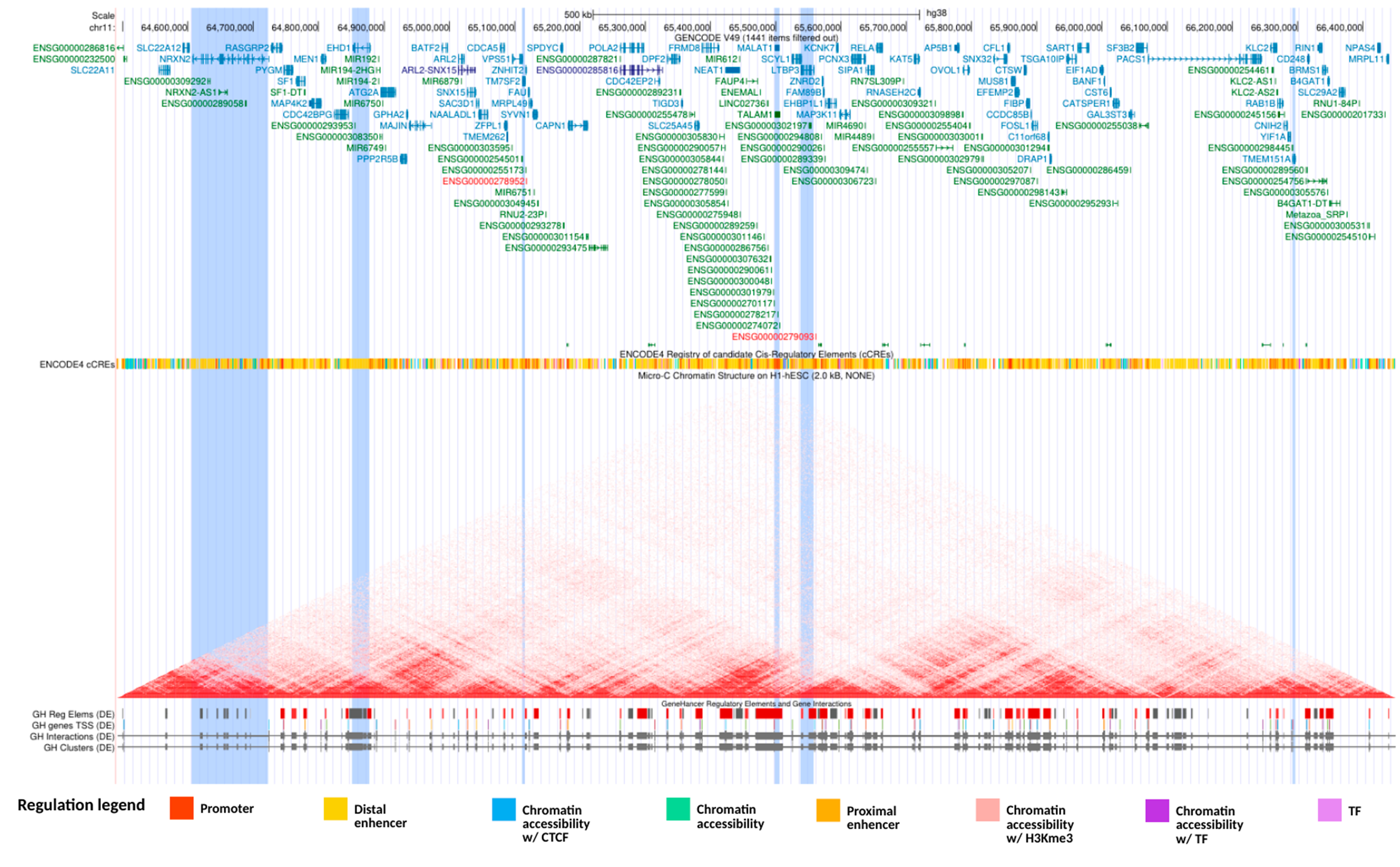

Supplement: Supplementary file 13 — Genomic region of MALAT1 locus. Genomic map obtained from UCSC Genome Browser and the displayed tracks are: GENCODE V49, ENCODE cCREs, GeneHancer and Hi-C and Micro-C (http://genome.ucsc.edu). MALAT1 and protein-coding genes with correlated expression are highlighted (PNG 1.71 MB) [file 12035_2026_5859_Fig13_ESM.png]

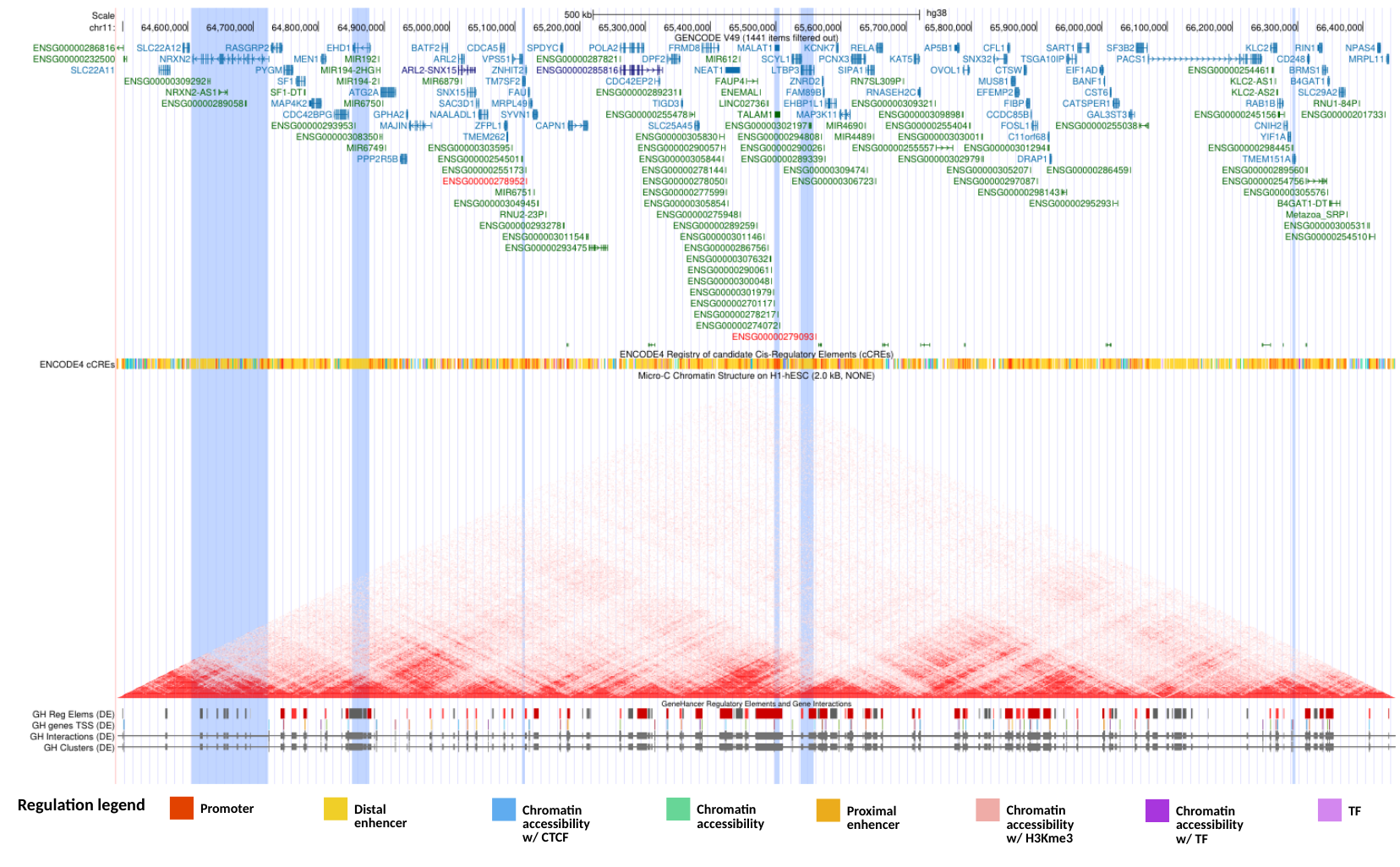

Supplement: Supplementary file 14 — High resolution image (TIFF 1.59 MB) [file 12035_2026_5859_MOESM7_ESM.tiff]
